# Prevalence of Subclinical Coronary Artery Atherosclerosis in the General Population

**DOI:** 10.1161/CIRCULATIONAHA.121.055340

**Published:** 2021-09-21

**Authors:** Göran Bergström, Margaretha Persson, Martin Adiels, Elias Björnson, Carl Bonander, Håkan Ahlström, Joakim Alfredsson, Oskar Angerås, Göran Berglund, Anders Blomberg, John Brandberg, Mats Börjesson, Kerstin Cederlund, Ulf de Faire, Olov Duvernoy, Örjan Ekblom, Gunnar Engström, Jan E. Engvall, Erika Fagman, Mats Eriksson, David Erlinge, Björn Fagerberg, Agneta Flinck, Isabel Gonçalves, Emil Hagström, Ola Hjelmgren, Lars Lind, Eva Lindberg, Per Lindqvist, Johan Ljungberg, Martin Magnusson, Maria Mannila, Hanna Markstad, Moman A. Mohammad, Fredrik H. Nystrom, Ellen Ostenfeld, Anders Persson, Annika Rosengren, Anette Sandström, Anders Själander, Magnus C. Sköld, Johan Sundström, Eva Swahn, Stefan Söderberg, Kjell Torén, Carl Johan Östgren, Tomas Jernberg

**Affiliations:** 1 Department of Molecular and Clinical Medicine (G. Bergström, E.B., O.A., B.F., O.H., A.R.), University of Gothenburg, Sweden.; 2 Institute of Medicine (M.B.), University of Gothenburg, Sweden.; 3 Department of Radiology, Institute of Clinical Sciences (J.B., E.F., A.F.), University of Gothenburg, Sweden.; 4 Sahlgrenska Academy, and School of Public Health and Community Medicine, Institute of Medicine (M.A., C.B.), University of Gothenburg, Sweden.; 5 Center for Health and Performance (M.B.), University of Gothenburg, Sweden.; 6 Occupational and Environmental Medicine/School of Public Health and Community Medicine (K.T.), University of Gothenburg, Sweden.; 7 Departments of Clinical Physiology (G. Bergström, O.H.), Region Västra Götaland, Gothenburg, Sweden.; 8 Cardiology (O.A.), Region Västra Götaland, Gothenburg, Sweden.; 9 Radiology (J.B., E.F., A.F.), Region Västra Götaland, Gothenburg, Sweden.; 10 Sahlgrenska University Hospital (M.B., B.F., A.R., K.T.), Region Västra Götaland, Gothenburg, Sweden.; 11 Department of Clinical Sciences (M.P., G. Berglund, G.E., M. Magnusson), Lund University, Malmö, Sweden.; 12 Experimental Cardiovascular Research, Clinical Research Center, Clinical Sciences Malmö (H.M.), Lund University, Malmö, Sweden.; 13 Departments of Internal Medicine (M.P.), Skåne University Hospital, Malmö, Sweden.; 14 Cardiology (M. Magnusson), Skåne University Hospital, Malmö, Sweden.; 15 Section of Radiology, Department of Surgical Sciences (H.A., O.D.), Uppsala University, Sweden.; 16 Cardiology (E.H.), Uppsala University, Sweden.; 17 Clinical Epidemiology (L.L., J.S.), Uppsala University, Sweden.; 18 Respiratory, Allergy and Sleep Research (E.L.), Uppsala University, Sweden.; 19 Department of Medical Sciences, and Uppsala Clinical Research Center (E.H.), Uppsala University, Sweden.; 20 Departments of Cardiology (J.A., E.S.), Linköping University, Sweden.; 21 Health, Medicine and Caring Sciences (J.A., E.S., J.E.E., F.H.N., C.J.Ö., A.P.), Linköping University, Sweden.; 22 Clinical Physiology (J.E.E.), Linköping University, Sweden.; 23 Radiology (A.P.), Linköping University, Sweden.; 24 CMIV, Centre of Medical Image Science and Visualization (J.E.E., A.P., C.J.Ö.), Linköping University, Sweden.; 25 Department of Public Health and Clinical Medicine, Medicine and Heart Centre (A.B., J.L., A. Sandström, A. Själander, S.S.), Umeå University, Sweden.; 26 Department of Surgical and Perioperative Sciences (P.L.), Umeå University, Sweden.; 27 Department of Clinical Science, Intervention and Technology (K.C.), Karolinska Institutet, Stockholm, Sweden.; 28 Unit of Cardiovascular and Nutritional Epidemiology, Institute of Environmental Medicine (U.d.F.), Karolinska Institutet, Stockholm, Sweden.; 29 Respiratory Medicine Unit, Department of Medicine Solna and Center for Molecular Medicine (M.C.S.), Karolinska Institutet, Stockholm, Sweden.; 30 Department of Clinical Sciences, Danderyd University Hospital (T.J.), Karolinska Institutet, Stockholm, Sweden.; 31 Department of Physical Activity and Health, The Swedish School of Sport and Health Sciences (GIH), Stockholm, Sweden (Ö.E.).; 32 Department of Endocrinology, Metabolism & Diabetes and Clinical Research Center, Karolinska University Hospital Huddinge, Stockholm, Sweden (M.E.).; 33 Department of Clinical Sciences Lund, Cardiology, Lund University and Skåne University Hospital, Lund, Sweden (D.E., M.A.M.).; 34 Department of Clinical Sciences Malmö (I.G.), Lund University and Skåne University Hospital, Lund, Sweden.; 35 Center for Medical Imaging and Physiology (H.M.), Lund University and Skåne University Hospital, Lund, Sweden.; 36 Department of Clinical Sciences Lund, Clinical Physiology (E.O.), Lund University and Skåne University Hospital, Lund, Sweden.; 37 Wallenberg Center for Molecular Medicine, Lund University, Sweden (M. Magnusson).; 38 North-West University, Hypertension in Africa Research Team (HART), Potchefstroom, South Africa (M. Magnusson).; 39 Heart and Vascular Theme, Department of Cardiology, and Clinical Genetics, Karolinska University Hospital, Stockholm, Sweden (M. Mannila).; 40 Department of Respiratory Medicine and Allergy, Karolinska University Hospital Solna, Stockholm, Sweden (M.C.S.).; 41 The George Institute for Global Health, University of New South Wales, Sydney, Australia (J.S.).

**Keywords:** coronary angiography, coronary artery disease, epidemiology, plaque, atherosclerotic, primary prevention, tomography

## Abstract

**Background:**

Early detection of coronary atherosclerosis using coronary computed tomography angiography (CCTA), in addition to coronary artery calcification (CAC) scoring, may help inform prevention strategies. We used CCTA to determine the prevalence, severity, and characteristics of coronary atherosclerosis and its association with CAC scores in a general population.

**Methods:**

We recruited 30 154 randomly invited individuals age 50 to 64 years to SCAPIS (the Swedish Cardiopulmonary Bioimage Study). The study includes individuals without known coronary heart disease (ie, no previous myocardial infarctions or cardiac procedures) and with high-quality results from CCTA and CAC imaging performed using dedicated dual-source CT scanners. Noncontrast images were scored for CAC. CCTA images were visually read and scored for coronary atherosclerosis per segment (defined as no atherosclerosis, 1% to 49% stenosis, or ≥50% stenosis). External validity of prevalence estimates was evaluated using inverse probability for participation weighting and Swedish register data.

**Results:**

In total, 25 182 individuals without known coronary heart disease were included (50.6% women). Any CCTA-detected atherosclerosis was found in 42.1%; any significant stenosis (≥50%) in 5.2%; left main, proximal left anterior descending artery, or 3-vessel disease in 1.9%; and any noncalcified plaques in 8.3% of this population. Onset of atherosclerosis was delayed on average by 10 years in women. Atherosclerosis was more prevalent in older individuals and predominantly found in the proximal left anterior descending artery. Prevalence of CCTA-detected atherosclerosis increased with increasing CAC scores. Among those with a CAC score >400, all had atherosclerosis and 45.7% had significant stenosis. In those with 0 CAC, 5.5% had atherosclerosis and 0.4% had significant stenosis. In participants with 0 CAC and intermediate 10-year risk of atherosclerotic cardiovascular disease according to the pooled cohort equation, 9.2% had CCTA-verified atherosclerosis. Prevalence estimates had excellent external validity and changed marginally when adjusted to the age-matched Swedish background population.

**Conclusions:**

Using CCTA in a large, random sample of the general population without established disease, we showed that silent coronary atherosclerosis is common in this population. High CAC scores convey a significant probability of substantial stenosis, and 0 CAC does not exclude atherosclerosis, particularly in those at higher baseline risk.

## Abstract 2

Supplemental Digital Content is available in the text.

**Clinical Perspective**

**What Is New?**

- Using coronary computed tomography angiography, we show that silent coronary atherosclerosis was common (42.1%), significant stenosis (≥50%) was less common (5.2%), and more severe forms were rarely found (1.9%) in a random sample of a middle-aged general population.
- Disease onset was delayed by 10 years in women. Higher prevalence of coronary atherosclerosis was observed with age and accumulation of risk factors.
- Coronary computed tomography angiography–detected atherosclerosis increased with increasing coronary artery calcification (CAC) score: All those with a high CAC score (>400) had atherosclerosis and 45.7% had significant stenosis, 5.5% of those with 0 CAC had atherosclerosis, and 0.4% had significant stenosis, with increasing prevalence at higher predicted risk.

**What Are the Clinical Implications?**

- We describe the prevalence and characteristics of atherosclerosis in the general population without established disease, which lays the foundation for developing and designing successful high-risk screening strategies.
- Although there is a strong association between high CAC score and significant stenosis, atherosclerosis is not excluded in those with 0 CAC, especially in those at high baseline risk.

**Editorial, see p 930**

Population strategies for prevention of cardiovascular disease have been successful and likely explain the reduction in the incidence of myocardial infarction (MI) in recent decades.^1^ However, MI is still a common condition, with high morbidity and a 28-day mortality of 24% in Sweden.^2^ Improved strategies are needed to identify individuals at the highest risk of cardiovascular disease to complement population-based efforts.^3^

Postmortem and intravascular ultrasound studies have shown that most acute coronary events coincide with a large and often ruptured atherosclerotic plaque in the coronary arteries.^4,5^ With recent advances in imaging technology,^6^ it is now possible to noninvasively visualize atherosclerotic plaques using coronary computed tomography angiography (CCTA)^7^ to identify individuals with subclinical coronary heart disease and thus at risk of cardiac events.^8^ Previous reports using CCTA to assess coronary atherosclerosis have been performed in selected populations, either as health evaluations^9^ or clinical evaluation of patients with chest pain,^10,11^ but not in the general population. Knowledge of the true prevalence and characteristics of atherosclerosis in the general population without established disease is a prerequisite for developing and designing successful high-risk screening strategies.

Imaging of coronary artery calcifications (CAC) using noncontrast computed tomography (CT) has been widely used to identify individuals with calcified coronary atherosclerosis, and there is a positive relationship between CAC and degree of CCTA-detected atherosclerosis.^12,13^ Although CAC scoring improves prediction of cardiac events beyond traditional risk scores in population studies,^14,15^ the CAC score does not provide information on the exact localization of coronary atherosclerosis, degree of stenosis, and presence of noncalcified plaques. Data from symptomatic patients suggest that CCTA may add discriminatory capacity for future coronary events beyond that of CAC.^16^ However, this finding remains to be corroborated and there are no published studies comparing CCTA and CAC data in a population-based prevention study.

SCAPIS (the Swedish Cardiopulmonary Bioimage Study), a general population-based prospective study, was designed to extensively characterize >30 000 men and women aged 50 to 64 years recruited at random from the population to obtain information that can be used to improve prevention strategies for cardiovascular disease.^17^ SCAPIS involves both an extensive imaging protocol including CCTA and CAC scoring as well as a comprehensive examination protocol. The aim of the current study was to determine the prevalence and burden of CCTA-detected coronary artery atherosclerosis and its association with CAC scores in the general population without established coronary heart disease. This is the first report from SCAPIS based on all 30 154 recruited participants.

## Methods

### Study Population

SCAPIS is a general population-based prospective study (www.scapis.org). Between 2013 and 2018, men and women aged 50 to 64 years were randomly recruited from the census register at 6 sites in Sweden (Gothenburg, Linköping, Malmö/Lund, Stockholm, Umeå, and Uppsala) and invited to a comprehensive examination as previously described.^17^ The multicenter study was approved by the ethical review board in Umeå (number 2010-228-31M). The participants gave written informed consent. Because of the sensitive nature of the personal data and study materials, they cannot be made freely available. However, by contacting the corresponding author or study organization (www.scapis.org), procedures for sharing data, analytic methods, and study materials for reproducing the results or replicating the procedure can be arranged following Swedish legislation.

Study inclusion is detailed in Figure I in the Data Supplement. Individuals were excluded from the present analyses if they did not undergo CCTA or if there was a technical failure in reading any of the 4 proximal segments (proximal right coronary, left main, proximal left anterior descending [LAD], and proximal circumflex artery) on the CCTA images. Individuals were also excluded if they had previously had a MI, percutaneous coronary intervention, or coronary artery bypass grafting. Information on previous MIs and cardiac procedures was self-reported (in the study questionnaire) or provided by at least 1 previous diagnosis of MI or a cardiac procedure on the Swedish inpatient register (National Board of Health and Welfare). MI in registry data was defined as I21 (according to International Classification of Diseases [ICD]–10) or 410 (according to ICD-8 or ICD-9). Cardiac procedures were defined as either percutaneous coronary intervention or coronary artery bypass grafting using the codes FNA-FNG (according to ICD-10) or 3066, 3105, 3127, 3158, 3092, or 3080 (according to ICD-8 or ICD-9). Individuals were excluded from the CAC analyses if they did not have readable CAC images (Figure I in the Data Supplement).

### Study Procedures

The study procedures have been described in detail elsewhere.^17^ In the present analyses, we used data from cardiac imaging, physical examinations, routine laboratory tests, accelerometry, a comprehensive questionnaire, and electronic health records reported to the Swedish inpatient register.

### Cardiac Imaging

Cardiac imaging in SCAPIS has been described in detail previously.^17^ Briefly, CT was performed using a dedicated dual-source CT scanner equipped with a Stellar Detector (Somatom Definition Flash, Siemens Medical Solutions). The CT scanners at each of the 6 sites were maintained using identical software and hardware throughout the study. CAC images were obtained using electrocardiogram-gated noncontrast CT imaging at 120 kV. In preparation for CCTA imaging, renal function was assessed and potential contraindications identified to exclude participants for whom administration of contrast media could pose a risk. A β-blocker (metoprolol) and sublingual glyceryl nitrate were given for control of heart rate and dilation of coronary arteries. The contrast medium iohexol (350 mg I/mL; GE Healthcare) was administered at a dose of 325 mg I/kg body weight. CCTA was performed at 100 or 120 kV using 5 different protocols depending on heart rate, heart rate variability, presence of calcifications, and body weight.

### Image Processing and Analyses

All noncontrast image sets were reconstructed (B35f HeartView medium CaScore) and CAC were identified and scored using the syngo.via calcium scoring software (Volume Wizard; Siemens). Lesions exceeding the calcium threshold of 130 Hounsfield units in at least 3 neighboring pixels in a volume of 1 mm^3^ were identified with 3D-based picking and viewing tools. The area of calcification of each 3-mm slice was multiplied with an intensity factor and summed across slices for the whole coronary artery tree to a CAC score according to Agatston.^18^ CAC scores (in Agatston Units) were further categorized into 0, 1 to 10 (ultralow), 11 to 100 (low), 101 to 400 (moderate), and >400 (high).

All contrast-enhanced CCTA image sets were reconstructed (I26f medium smooth advanced smoothing algorithm) and visually scored for atherosclerosis using syngo.via software. All readers (36 trained thoracic radiologists or cardiologists) had between 1 and >10 years of training in reading CCTA and a competence level 1 (11 readers, 11.0% of all reads), level 2 (16 readers, 55.6% of all reads), or level 3 (9 readers, 33.4% of all reads) in accordance with the American College of Cardiology Foundation/American Heart Association Clinical Competence Statement on cardiac CT.^19^ Readers at the 6 sites visually read the CCTA image datasets and consecutively entered information into the SCAPIS electronic case report form. The readers attended yearly training and information sessions to assure consistency in reading and reporting.

For reporting coronary atherosclerosis from CCTA, we used the 18 coronary segment model defined by the Society of Cardiovascular Computed Tomography.^20^ To streamline reading and increase quality of the most important findings, readers focused on the 11 clinically most relevant segments (segments 1 through 3, 5 through 7, 9, 11 through 13, and 17), which were compulsory to report; the remaining segments were only reported if they had atherosclerosis or calcium blooming. The coronary segments were visually examined for the presence of plaques and each plaque was visually characterized as calcified or noncalcified. We use the phrase “only noncalcified plaques” for participants who had exclusively noncalcified plaques in all affected segments and “any plaque noncalcified” for participants who had either only noncalcified plaques or a mix of noncalcified and calcified plaques. Per-segment status of the coronary vessel was defined as follows: no atherosclerosis, 1% to 49% stenosis; ≥50% (ie, significant) stenosis; not assessable because of calcium blooming (see following); or not assessable because of technical failure (see following) or segment missing. Luminal obstruction was defined by visually estimating diameter stenosis (using the average of the longest and shortest diameter at the site of stenosis).

Defining the level of stenosis from calcified plaques is a challenge for CCTA and calcifications are known to overestimate the level of stenosis.^21,22^ If calcium content of the segment was severe and precluded judgment of level of stenosis, the segment was classified as having a calcium blooming artifact and, in the main analyses, the level of stenosis was set to 1% to 49%. In a sensitivity analysis presented in the Data Supplement, the level of stenosis for a calcium blooming artifact was set to ≥50%.

Technical artefacts (eg, owing to motion, stair-step, beam-hardening, reduced signal-to-noise, low vessel contrast intensity) of a magnitude that made judgment of segment status invalid were reported as technical failures when reading the segment and coded as missing data in analyses of coronary atherosclerosis prevalence. Details on calcium blooming, technical failures, reproducibility, and consistency across sites are presented in the Data Supplement.

Participants with ≥50% stenosis were classified as having 1-, 2-, or 3-vessel disease and whether they had left main or proximal LAD disease. Segments with noncalcified plaques were also identified.

### Risk Factor Burden

The extent of CCTA-detected atherosclerosis was compared with risk factor burden using both the Systematic Coronary Risk Evaluation (SCORE)^23^ and the pooled cohort equation (PCE).^24^ The following risk categories were used: for SCORE, low (<2%), moderate (2% to 5%), and high (>5%) 10-year risk of atherosclerotic cardiovascular disease (fatal); and for PCE, low (<5%), borderline (≥5 to <7.5%), intermediate (≥7.5 to <20%), and high (≥20%) 10-year risk of atherosclerotic cardiovascular disease (nonfatal/fatal). The extent of CCTA-detected atherosclerosis in relation to risk factor burden assessed by PCE was also determined in those with a CAC score of 0.

### Statistical Analysis

Data were analyzed without imputations and we estimated the prevalence of coronary atherosclerosis in the general population based on individuals who agreed to participate in SCAPIS. As a sensitivity analysis to test the external validity of this estimate, we standardized the SCAPIS sample to 2 target populations of interest using sociodemographic register data^25^: the age-matched population in the SCAPIS catchment areas, which reflects the population that was invited to participate in SCAPIS; and the age-matched population of Sweden as a whole using inverse probability for participation weighting (see the Data Supplement).

The delayed onset of CCTA-detected atherosclerosis (1% to 49% stenosis and ≥50% stenosis) in women versus men was modeled to identify the minimal value of the sum of the squared differences between the annualized prevalence in women and men (see the Data Supplement).

Logistic regression was used to test which factors were associated with discrepancies between CCTA-detected atherosclerosis and CAC scores. Factors included in the logistic regression were sex, age, obesity (body mass index >30), current smoker, cholesterol-lowering medication, antihypertensive medication, diabetes (self-reported), systolic blood pressure, total cholesterol, and high-density lipoprotein cholesterol.

Reporting of this study follows the recommendations of the STROBE statement (Strengthening the Reporting of Observational Studies in Epidemiology).^26^ Data were analyzed by authors M.A., C.B., and E.B.

## Results

### Population

In total, 30 154 individuals were recruited to SCAPIS (characteristics presented in Table I in the Data Supplement). The overall participation rate of those invited was 50.3% and was similar between ages and sexes (Table II in the Data Supplement). Of the total number recruited, 29 613 underwent CT and 27 385 underwent CCTA. Characteristics of participants who underwent CT but not CCTA (n=2228) are described in Table III in the Data Supplement. The main reasons for not undergoing CCTA were issues with the safety of the participant (44%), unwillingness to be exposed to the examination (24%), or issues with venous access (17%; details in Table IV in the Data Supplement). The median effective radiation dose from CAC imaging and CCTA imaging was 0.34 mSv (interquartile range, 0.29 to 0.48) and 1.33 mSv (interquartile range, 0.95 to 1.77), respectively.

After excluding 1805 individuals because image quality of their proximal segments was poor and a further 398 individuals because they had previous MI or coronary intervention, 25 182 participants remained for the analysis of CCTA-detected atherosclerosis (characteristics in Table 1, missing data in Table V in the Data Supplement). Results on reading consistency of CCTA images are presented in Table VI in the Data Supplement, and Figures II and III in the Data Supplement. Technical artefacts making judgment of segment status invalid are reported in Table VII in the Data Supplement. Characteristics of individuals with technical failures in any of the 4 most proximal segments are described in Table VIII in the Data Supplement.

**Table 1.**
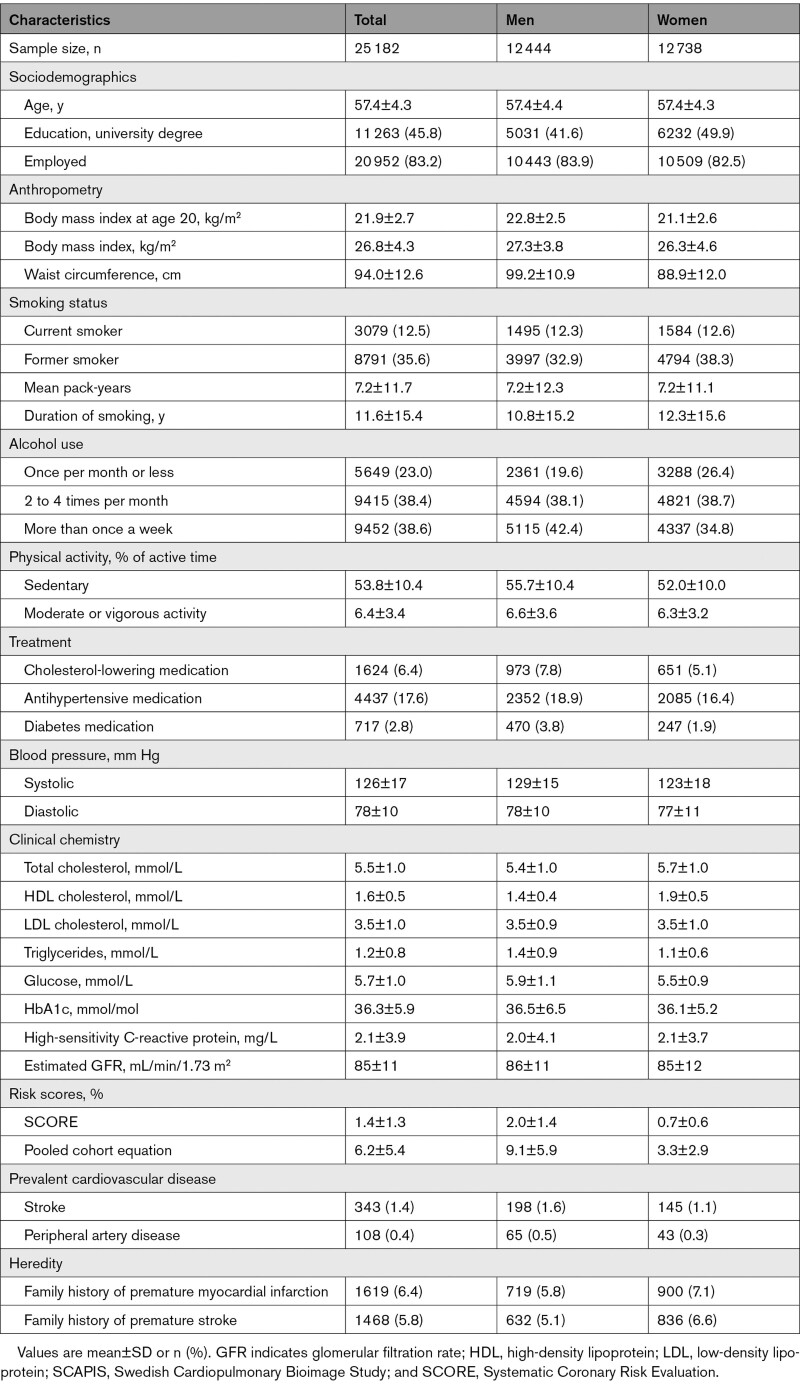
Characteristics of SCAPIS Participants Without Established Coronary Heart Disease Who Underwent Successful Coronary Computed Tomography Angiography

After excluding a further 168 individuals who did not have readable CAC images, 25 014 participants were included in the combined analyses of CCTA and CAC (Figure I in the Data Supplement).

### Prevalence and Characteristics of CCTA-Detected Atherosclerosis

**Figure 1.**
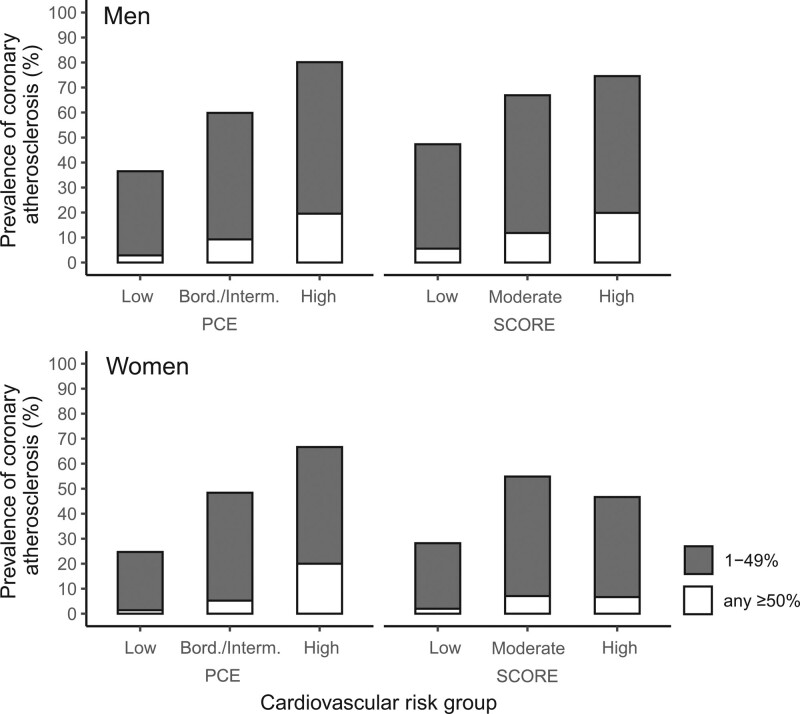
**Prevalence of coronary computed tomography angiography–detected atherosclerosis by sex and category of risk score.** Prevalence of coronary computed tomography angiography–detected coronary atherosclerosis and degree of stenosis in the SCAPIS cohort (Swedish Cardiopulmonary Bioimage Study; n=25 182) divided by sex and category of cardiovascular risk according to pooled cohort equation (PCE; low <5%, borderline/intermediate ≥5 to <20%, and high ≥20% 10-year risk of atherosclerotic cardiovascular disease [fatal/nonfatal]) and Systematic Coronary Risk Evaluation (SCORE; low <2%, moderate 2% to 5%, and high >5% 10-year risk of atherosclerotic cardiovascular disease [fatal]).

Of the 25 182 participants in SCAPIS who underwent a technically successful CCTA and had not previously experienced a MI or coronary intervention, any CCTA-detected atherosclerosis was found in 42.1% (95% CI, 41.3–42.7%) and any significant stenosis (≥50%) in 5.2% (95% CI, 4.9–5.5%; Table 2). Severe forms of coronary atherosclerosis were less common, with significant stenosis in left main, proximal LAD, or 3-vessel disease found in 1.9% (95% CI, 1.7–2.0%) of the population (Table 2). Prevalence of atherosclerosis was 1.9 times higher in men than in women and increased sharply with age in both sexes (being 1.8 times higher in the upper versus lower age range [60 to 64 versus 50 to 54 years] in the combined population; Table 2 and Figure IV in the Data Supplement). Modeling showed that the delay in disease onset in women compared with men was ≈10 years for both 1% to 49% stenosis and ≥50% stenosis (Figure V in the Data Supplement). Prevalence of atherosclerosis increased with risk factor burden, being 2.1and 2.9 times more common in participants at high versus low risk according to SCORE and PCE, respectively (Figure 1). The prevalence of atherosclerosis was not higher in women at high versus moderate risk according to SCORE (Figure 1), but only 15 women were in the high-risk SCORE category. When the reported prevalence was adjusted to the age-matched population in the SCAPIS catchment areas, which reflects the population that was invited to participate in SCAPIS or Sweden as a whole using inverse probability for participation weighting, the estimates changed only marginally (Table IX in the Data Supplement).

**Table 2.**
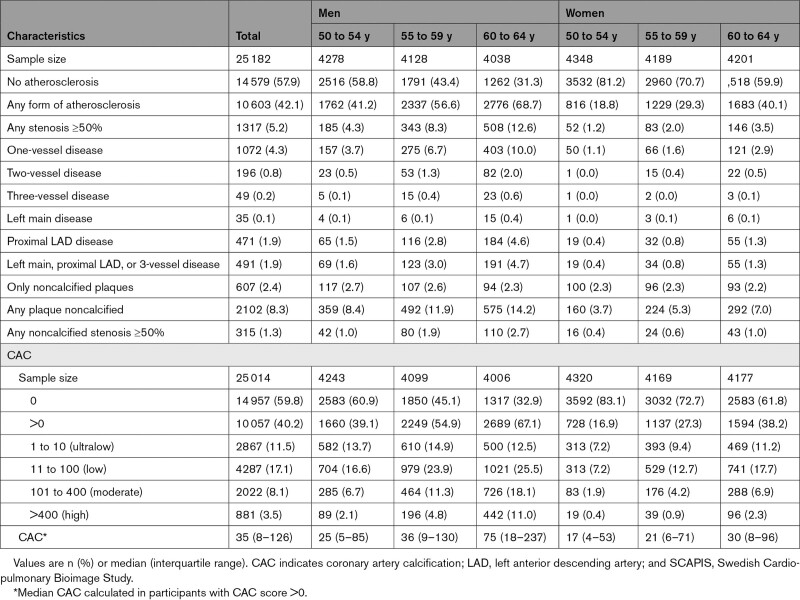
Prevalence of Coronary Computed Tomography Angiography–Detected Atherosclerosis and CAC in SCAPIS Participants Without Established Coronary Heart Disease Who Underwent Successful Coronary Computed Tomography Angiography (n=25 182) and Coronary Computed Tomography Angiography and CAC Scoring (n=25 014), Divided by Sex and Age

In the analyses presented in the main text, calcium blooming was set to 1% to 49% stenosis. When calcium blooming was set to ≥50% stenosis, the prevalence of any significant stenosis increased from 5.2% to 9.3% (Table X in the Data Supplement) and severe forms of coronary atherosclerosis with significant stenosis in left main, proximal LAD, or 3-vessel disease increased from 1.9% to 3.7% of the population.

**Figure 2.**
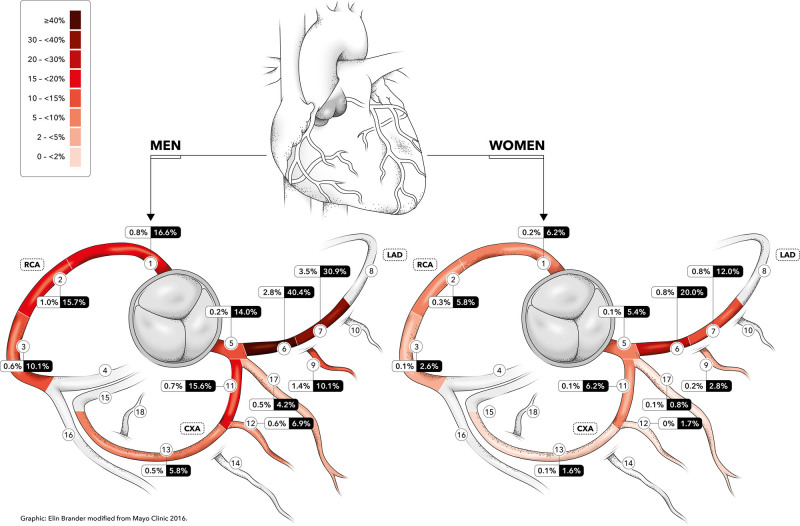
**Distribution of coronary computed tomography angiography–detected atherosclerosis.** Frequency of atherosclerosis in the 11 most proximal coronary segments in men (n=12 444) and women (n=12 738) in the SCAPIS cohort (Swedish Cardiopulmonary Bioimage Study). The heat map refers to the frequency of any form of coronary computed tomography angiography–detected atherosclerosis. The numbers within boxes indicate the frequency of different degrees of vessel stenosis (white box, ≥50% stenosis; black box, any form of coronary computed tomography angiography–detected atherosclerosis). Figure modified from Ayoub et al.,^52^ used with permission of Mayo Foundation for Medical Education and Research, all rights reserved.

**Figure 3.**
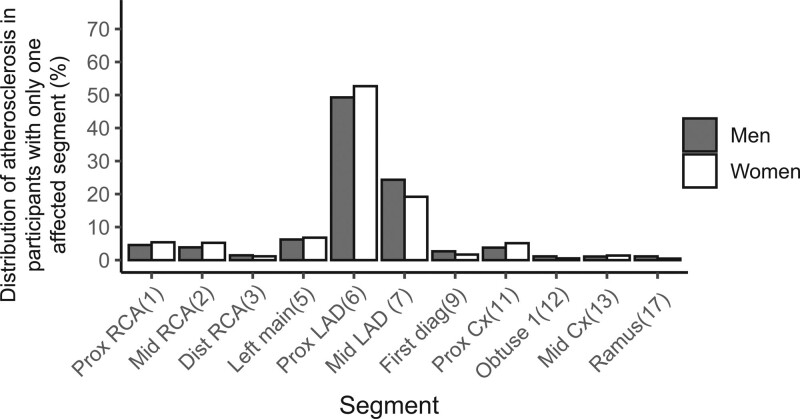
**Segmental distribution of coronary computed tomography angiography–detected atherosclerosis in participants of the SCAPIS cohort (Swedish Cardiopulmonary Bioimage Study) with only 1 affected segment (n=3867).** Segment numbers according to the Society of Cardiovascular Computed Tomography.^20^ Segment numbers are indicated in parentheses. Cx indicates circumflex artery; LAD, left anterior descending artery; and RCA, right coronary artery.

Atherosclerosis was distributed across all segments with a predilection for proximal segments (most commonly found in proximal and mid LAD), with no apparent differences in the distribution between sexes or age groups (Figure 2 and Table XI in the Data Supplement). The same distribution was found in participants in the early developmental phase of atherosclerosis (ie, those with only 1 affected coronary segment), with proximal LAD most often involved (Figure 3).

Participants with only noncalcified plaques were rare (2.4%) whereas a mix of 1 or more noncalcified plaques together with calcified plaques was observed in 8.3% of the population (Table 2).

### Association Between CCTA-Detected Atherosclerosis and CAC Scores

In general, the prevalence of CAC in the cohort with both valid CCTA and CAC images (n=25 014) followed the same pattern as seen for CCTA-detected atherosclerosis, with 40.2% of the population being CAC-positive and a higher prevalence with age and in men (Table 2). Most CAC-positive participants had low (11 to 100) or ultralow (1 to 10) CAC scores (Table 2). In those who were CAC-positive, the median CAC score was 35 (interquartile range, 8 to 126; Table 2).

The prevalence of any CCTA-detected atherosclerosis, any significant stenosis, and more severe forms of CCTA-detected atherosclerosis increased with increasing CAC categories, both in men and women (Table 3). In participants with 0 CAC or CAC score 1 to 10, 5.5% and 81.9% had CCTA-detected atherosclerosis, respectively. In participants with CAC score 11 to 100, 7.0% had at least 1 significant stenosis and 14.9% had noncalcified plaques. In those with CAC score 101 to 400, 24% had at least 1 CCTA-identified significant stenosis and 8.5% had left main, proximal LAD, or 3-vessel disease. In participants with a high CAC score (>400), 54.3% did not have any significant stenosis; 20.3% had CCTA-detected left main, proximal LAD, or 3-vessel disease.

**Table 3.**
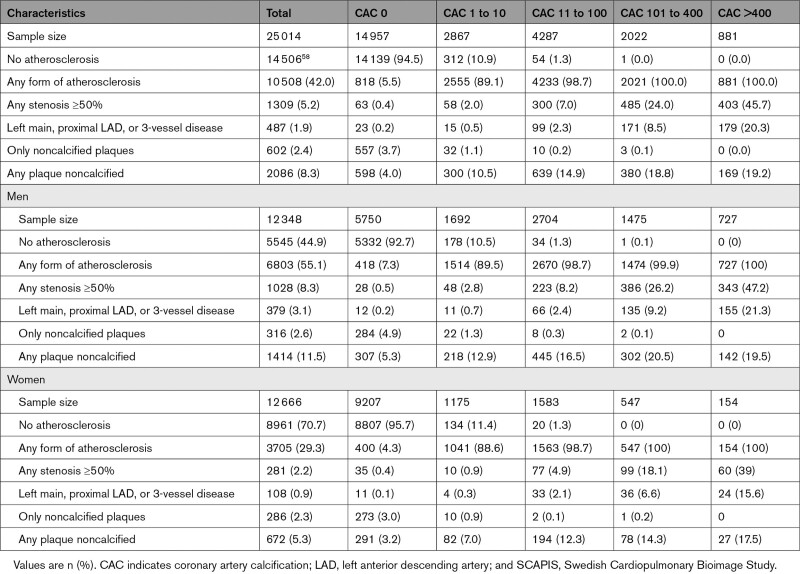
Prevalence of Coronary Computed Tomography Angiography–Detected Atherosclerosis in SCAPIS Participants Without Established Coronary Heart Disease Who Underwent Both Successful Coronary Computed Tomography Angiography and CAC Scoring (n=25 014), Divided by Sex and CAC Category

### Complementary Information Provided by CCTA

**Figure 4.**
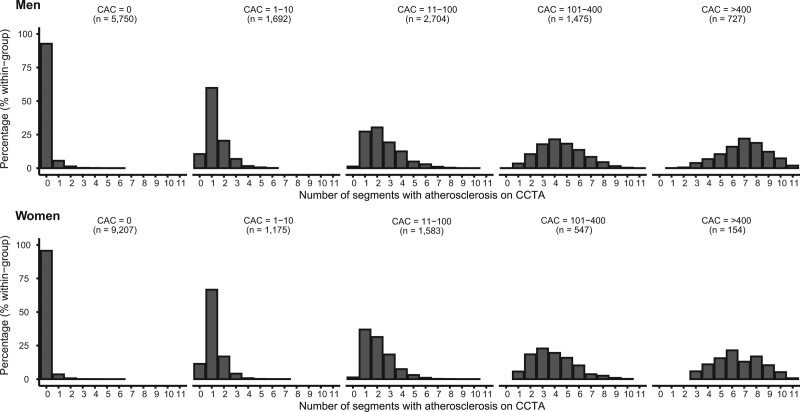
Distribution of the number of coronary artery segments with coronary computed tomography angiography (CCTA)–detected atherosclerosis, divided by coronary artery calcification (CAC) category and sex.

The number of coronary segments with CCTA-detected atherosclerosis showed a large variation within each CAC score category (Figure 4). In those with CAC score 1 to 10, 29.7% of men and 22.0% of women had CCTA-detected atherosclerosis in 2 or more coronary segments. In participants with CAC score 11 to 100, 41.1% of men and 30.3% of women had CCTA-detected atherosclerosis in 3 or more coronary segments.

A number of participants (n=367) did not have CCTA-detected atherosclerosis despite having CAC scores >0. The majority of these (n=312) had ultralow CAC scores (1 to 10) and the scores were mainly in the lower range of this category (median, 1; interquartile range, 1 to 3). In participants with ultralow CAC scores, body mass index was slightly higher in those without CCTA-detected atherosclerosis than in those with CCTA-detected atherosclerosis (body mass index 27.6 and 27.2, respectively; *P*=0.058). A few participants had only noncalcified plaques on CCTA but had CAC scores 1 to 10 (n=32), 11 to 100 (n=10), and even 101 to 400 (n=3). In the cohort with both valid CCTA and CAC images, the number of participants with 0 CAC but CCTA-detected plaques was 818 (3.3%), slightly higher than the number with only noncalcified plaques on CCTA (n=602, 2.4%; Table 3).

The risk profile of participants with 0 CAC but CCTA-detected atherosclerosis (n=818) was between that of participants with 0 CAC and no CCTA-detected atherosclerosis and that of participants with CAC scores >0 and CCTA-detected atherosclerosis (Table XII in the Data Supplement). In the population with 0 CAC, the participants with CCTA-detected atherosclerosis (n=818) were more often male, had a higher age, were more likely to be smokers, had higher cholesterol and lower high-density lipoprotein, and higher blood pressure, and were more often obese compared with participants without CCTA-detected atherosclerosis (Tables XII and XIII in the Data Supplement). These risk factors (with the exception of current smokers) were also more common in participants with a CAC score >0 (n=367) versus those with 0 CAC in the population without CCTA-detected atherosclerosis (Tables XII and XIII in the Data Supplement).

Of the population with 0 CAC, CCTA-detected atherosclerosis was found in 6.0% of participants with a strong family history of MI, 6.8% of participants who were current smokers, and 8.1% of participants receiving treatment for diabetes.

The proportion of participants with 0 CAC but CCTA-detected atherosclerosis increased with increasing categories of the PCE and was 15% in the highest PCE risk category (Table 4). In participants with 0 CAC and borderline and intermediate risk according to PCE, the proportion of participants with CCTA-detected atherosclerosis was 5.3% and 9.2%, respectively.

**Table 4.**
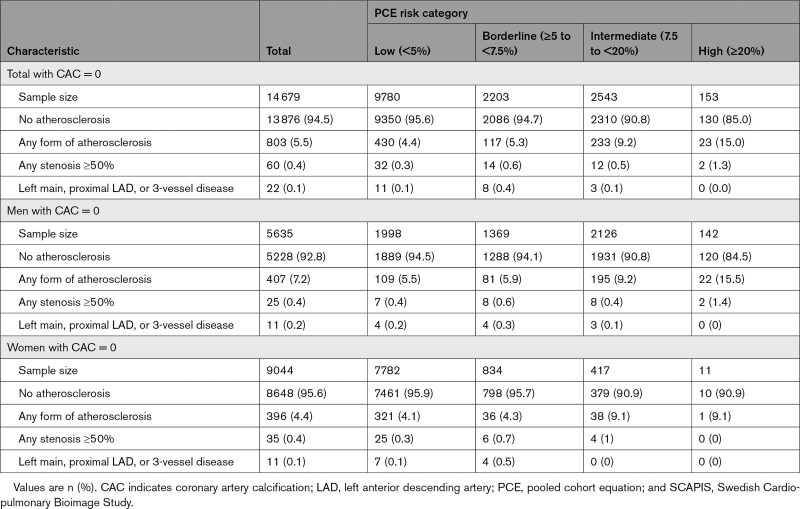
Prevalence of Coronary Computed Tomography Angiography–Detected Atherosclerosis in SCAPIS Participants Without Established Coronary Heart Disease Who Underwent Both Successful Coronary Computed Tomography Angiography and CAC Scoring, for Whom PCE Could Be Calculated, and With 0 CAC (n=14 679), Divided by Sex and PCE Risk Group

## Discussion

This study used CCTA in a large, random sample of the general population. In a middle-aged population without previous MI or coronary intervention, silent coronary atherosclerosis was common, with 42.1% of participants having plaques in their coronary arteries. Significant stenosis (≥50%) was less common (in 5.2% of the population) and more severe forms of coronary atherosclerosis such as left main, proximal LAD, or 3-vessel disease were rarely found (in only 1.9% of the population). A higher prevalence of coronary atherosclerosis was observed in men and with higher age and accumulation of risk factors. CCTA-detected atherosclerosis increased with increasing CAC score category but subgroups of participants could be identified whose coronary atherosclerosis was not accurately represented by their CAC score.

Previous data on the prevalence of coronary atherosclerosis in the general population are rare. A number of autopsy studies have reported the prevalence of coronary atherosclerosis, but these studies are often in small and highly selected populations and use a variety of reporting systems, ^27^ making comparisons with the general population difficult. Several population-based imaging studies have used CT to quantify CAC as a surrogate marker of atherosclerosis and showed a prevalence of a positive CAC between 50% and 89% dependent on age.^14,28–30^ However, no estimates of coronary artery stenosis can be derived from these studies.^31^ Earlier reports using CCTA, which have shown prevalence of atherosclerosis ranging between 22% and 43% and significant stenosis at 5% to 8%,^9,32^ are from selected populations only. True population estimates of prevalence are of importance if we are to design and apply successful screening strategies.^33^ Using inverse probability for participation weighting, we showed that our population was representative of the age-matched background population with only minimal selection bias. Thus, the prevalence of CCTA-detected coronary atherosclerosis in the general population reported in our study closely reflects the situation in Sweden. According to disease statistics from Europe and the United States,^34,35^ Sweden has an incidence of cardiovascular disease similar to that of many other western countries. In addition, we confirmed a 10-year delay in the development of both nonobstructive (1% to 49% stenosis) and obstructive (≥50% stenosis) silent coronary atherosclerosis in women, in agreement with earlier estimates of a 10-year difference between men and women in the onset of atherosclerosis-related clinical cardiovascular disease.^23,24^

Our results show that proximal LAD was the most common location for plaques, corroborating early autopsy studies^36,37^ and suggesting that atherosclerosis has a predilection for proximal branching sites perhaps with turbulent flow patterns.^38,39^ Proximal LAD was also the most common location of significant stenosis. Although the cross-sectional design of our study prevents us from showing how atherosclerosis develops over time, we identified individuals with only 1 affected coronary segment who are likely in the early developmental phase of coronary atherosclerosis. We observed that proximal LAD was also the most common location of atherosclerosis in these individuals.

Noncalcified plaques have been suggested as a sign of a more vulnerable form of coronary atherosclerosis prone to cause events, although their role in causing cardiovascular events needs to be established.^7^ Whereas the prevalence of participants with only noncalcified plaques on CCTA was low (2.4%), the percentage of participants with noncalcified plaques mixed among plaques with calcifications was higher and 14.2% of men aged 60 to 64 years had noncalcified plaques. However, it is well-known that not all plaques will lead to clinical events, and future longitudinal studies are required to determine whether the presence of noncalcified plaques confers an increased risk of future events.

In this large population study comparing CCTA and CAC data, we observed a positive association between CCTA-detected atherosclerosis and CAC scores, consistent with previous observations.^40,41^ In participants with a high CAC score (>400), the frequencies of coronary stenosis ≥50% and very severe forms of coronary atherosclerosis were 45.7% and 20.3%, respectively. However, there were discrepancies between CCTA and CAC data. In participants with an ultralow CAC score (1 to 10), 22.0% of women and 29.7% of men had 2 or more coronary segments with CCTA-detected atherosclerosis, and 7.0% of participants with a low CAC score (11 to 100) had coronary stenosis ≥50%. It was also evident that CCTA and CAC imaging provided complementary data on coronary atherosclerosis because not all calcifications detected using CAC scoring were verified as CCTA-detected atherosclerosis by the readers. This was mainly seen in participants with ultralow CAC score (1 to 10) and could be explained by difficulties in assessing such low CAC scores with high reproducibility^42^ and the well-known difficulties in identifying small calcifications in the presence of iodinated contrast media.^43^ Furthermore, CCTA-detected atherosclerosis in participants with 0 CAC was sometimes reported as calcified from CCTA images. These differences could in some cases be attributable to miscoding but most likely result from differences in the methodology to detect calcification.^44^ If we define noncalcified plaques as atherosclerosis in participants with 0 CAC, as would be the situation in a CAC screening program, the prevalence of participants with only noncalcified plaques would be 3.3%, higher than the prevalence of all noncalcified plaques detected using CCTA (2.4%).

A CAC score of 0 is generally considered a very good prognosis.^40^ Recent US guidelines state that the CAC score could be used in primary prevention to improve classification of individuals identified by PCE to be at intermediate 10-year risk of atherosclerotic cardiovascular disease; a CAC score of 0 would indicate a lowered risk and therefore not favor treatment with statins, which would otherwise be recommended in this group.^45^ However, studies and guidelines have questioned the negative predictive value of 0 CAC because significant atherosclerosis in the absence of CAC is possible.^46^ In the age group recruited to the current study (50 to 64 years), a large proportion would be expected to have 0 CAC (and here we report 60%). Of the group with 0 CAC in our study, 5.5% (4.3% of women and 7.3% of men) had CCTA-detected atherosclerosis in their coronary arteries. The risk factor burden in the group with 0 CAC but CCTA-detected atherosclerosis was generally higher than in the group with 0 CAC and no CCTA-detected atherosclerosis. In our population with 0 CAC and at intermediate risk as defined by PCE, 9.2% had CCTA-detected atherosclerosis, and would thus have been misclassified (according to US guidelines^45^) as having a lower than intermediate risk. The US guidelines do not recommend using 0 CAC to indicate a lower risk when the risk factor burden is large and the likelihood of noncalcified plaques could be high.^45^ Indeed, we showed that CCTA-detected atherosclerosis was present in a subset of our population with a CAC score of 0 who were currently smokers (6.8%), had a strong family history of MI (6.0%), or had diabetes (8.1%). Data from SCOT-HEART (Scottish Computed Tomography of the HEART Trial)^47^ and PROMISE (Prospective Multicenter Imaging Study for Evaluation of Chest Pain)^48^ suggest that CCTA-detected coronary atherosclerosis, irrespective of degree of stenosis, is an important driver of clinical events in symptomatic populations. Follow-up studies are required to determine whether CCTA-detected coronary atherosclerosis is associated with similar risk in an asymptomatic population.

This study has limitations. Because this is a large-scale study in the general population, we used stringent safety criteria, including use of low-dose radiation, avoiding repetition of nondiagnostic scans, and strict risk management for contrast injections, which resulted in a relatively high exclusion rate. Although we successfully scanned and obtained high-quality CCTA images from 86% of the SCAPIS population, a more aggressive imaging strategy could be applied in a real-world screening situation. The readers had access to both contrast and noncontrast image sets and the readings of CCTA and CAC data are therefore not independent.

In conclusion, our study showed that (1) silent coronary atherosclerosis is common in the general population without established disease, (2) coronary atherosclerosis detected using CCTA is associated with CAC scores, and (3) subgroups of participants could be identified whose coronary atherosclerosis was not accurately represented by their CAC score, showing a potential additive value of CCTA imaging.

## Acknowledgments

The authors thank Dr Rosie Perkins (Institute of Medicine, University of Gothenburg) for editing the manuscript. The study organization is acknowledged in the Data Supplement.

## Sources of Funding

This study received funding from the Swedish Heart-Lung Foundation, Knut and Alice Wallenberg Foundation, Swedish Research Council and Vinnova (Sweden’s Innovation agency), University of Gothenburg and Sahlgrenska University Hospital, Karolinska Institutet and Stockholm county council, Linköping University and University Hospital, Lund University and Skåne University Hospital, Umeå University and University Hospital, and Uppsala University and University Hospital.

## Disclosures

Dr Ahlström is a founder and employee of Antaros Medical AB. Dr Hagström received research grant funding from Pfizer (significant), Amgen (significant), and DalCor National coordinator (modest), and consulting or honoraria from AstraZeneca, Amgen, Sanofi-Aventis, Novartis, and NovoNordisk (modest). Dr Sundström reports stock ownership in companies providing services to Itrim, Amgen, Janssen, Novo Nordisk, Eli Lilly, Boehringer, Bayer, Pfizer, and AstraZeneca. Dr Söderberg reports speakers honoraria and advisory board participation for Actelion Ltd. The other authors report no conflicts.

## Supplemental Materials

Methods

Data Supplement Figures I–V

Data Supplement Tables I–XIII

## Supplementary Material


