# Supplementary material for: Prevalence of Subclinical Coronary Artery Atherosclerosis in the General Population
Source: Circulation. 2021 Sep 21;144(12):916–29. doi: 10.1161/CIRCULATIONAHA.121.055340 (PMC8448414; doi:10.1161/CIRCULATIONAHA.121.055340)
Supplement: Supplementary file 1 [file cir-144-916-s001.pdf]

SUPPLEMENTAL MATERIAL

Supplement to: Bergström G, Persson M, Adiels M, et al. Prevalence of subclinical coronary artery atherosclerosis in the general population

Contents

STUDY ORGANIZATION 3

Study governance (active and former) 3

Study operations 4

Study execution 5

METHODS 7

Reproducibility and consistency of reading CCTA across readers and sites 7

Definitions of CCTA scores used to calculate burden of coronary atherosclerosis per subject 7

Inverse probability for participation weighting (IPPW) 7

Handling of calcium blooming 8

SUPPLEMENTARY FIGURES 9

Supplemental Figure I: Flow diagram of study inclusion. 9

Supplemental Figure II: Atherosclerosis per subject for individual readers, stratified by CACS category. 10

Supplemental Figure III: Atherosclerosis per subject for each site, stratified by CACS category. 11

Supplemental Figure IV: Prevalence of CCTA-detected atherosclerosis by sex and age. 12

Supplemental Figure V: Modeling of delayed onset of coronary atherosclerosis in women. 13

SUPPLEMENTARY TABLES 14

Supplemental Table I. Characteristics of study participants in the full SCAPIS cohort. 14

Supplemental Table II: Participation rate in SCAPIS per site, age and sex. 15

Supplemental Table III: Characteristics of subjects who underwent CT but not CCTA. 16

Supplemental Table IV: Reasons for not performing CCTA in subjects who underwent CT. 17

Supplemental Table V: Missing data in participants in SCAPIS without established CHD who underwent successful CCTA (n=25,182). 18

Supplemental Table VI. Inter- and intra-reader reproducibility for 10 coronary segments. 19

Supplemental Table VII: Technical failures and calcium blooming artefacts in the 11 central coronary vascular segments. 20

Supplemental Table VIII: Characteristics of subjects with technical failures in any of the 4 most proximal segments. 21

Supplemental Table IX: Prevalence of atherosclerosis in the SCAPIS cohort after adjustment to age-matched populations using IPPW. 22

Supplemental Table X: Prevalence of atherosclerosis in the SCAPIS cohort when calcium blooming was defined as ≥50% stenosis. 23

Supplemental Table XI. Distribution of CCTA-detected atherosclerosis in the 11 most proximal coronary segments in men and women divided by age group in the SCAPIS cohort (n=25,182). 24

Supplemental Table XII: Characteristics of participants in SCAPIS without established CHD who underwent both successful CCTA and CAC scoring (n=25,014), divided by zero or positive CAC and absence or presence of CCTA-detected atherosclerosis. 26

Supplemental Table XIII. Odds ratios (95% confidence intervals) for risk factors associated with discrepancies between CCTA-detected atherosclerosis and CAC scores in participants in SCAPIS without established CHD who underwent both successful CCTA and CAC scoring. 28

STUDY ORGANIZATION

Study governance (active and former)

National Steering Committee
Directors:
Göran Bergström, University of Gothenburg, Director
Carl Johan Östgren, Linköping University, vice Director

Members (active and former):
Anders Blomberg, Umeå University, Umeå; **John Brandberg,** University of Gothenburg, Gothenburg; **Carl-Johan Carlhäll**, Linköping University, Linköping; **Kerstin Cederlund,** Karolinska Institutet, Stockholm; **Gunnar Engström,** Lund University, Lund; **Jan Engvall,** Linköping University, Linköping; **Mats G. Hansson,** Uppsala University, Uppsala; **Tomas Jernberg,** Karolinska Institutet, Stockholm; **Lars Lind,** Uppsala University, Uppsala; **Eva Lindberg,** Uppsala University, Uppsala; **Margaretha Persson,** Lund University, Lund; **Fredrik Nyström**, Linköping University, Linköping; **Annika Rosengren,** University of Gothenburg, Gothenburg; **Magnus Sköld,** Karolinska Institutet, Stockholm; **Johan Sundström,** Uppsala University, Uppsala; **Stefan Söderberg,** Umeå University, Umeå; **Kjell Torén,** University of Gothenburg, Gothenburg

Coordinator:
Bim Boberg, the Swedish Heart and Lung Foundation, Stockholm

The Swedish CArdioPulmonary bioImage Study (SCAPIS) is a collaborative project between the following Swedish Universities and Swedish University Hospitals:
University of Gothenburg and Sahlgrenska University Hospital; Karolinska Institutet and Karolinska University Hospital; Linköping University and Linköping University Hospital; Lund University and Skåne University Hospital; Umeå University and University Hospital of Umeå; Uppsala University and Uppsala University Hospital.

Scientific Advisory Board
Marike Boezen, University of Groningen, Groningen, NL; **Göran Berglund,** Skåne University Hospital and Lund University, Malmö, SE; **Robert Clarke,** University of Oxford, UK; **Marc Dewey,** Radiology Institute at Charité University of Medicine, Berlin, GE; **Ulf De Faire,** Karolinska Institutet, Solna, SE; **Kay-Tee Khaw,** University of Cambridge, UK; **Juhani Knuuti,** Turku PET Centre, Turku University Hospital, Turku, FI; **Claes-Göran Löfdahl,** Skåne University Hospital and Lund University, Lund, SE; **Eva Prescott,** University of Copenhagen and Bispebjerg University Hospital, Copenhagen, DK; **Anders Waldenström,** Umeå University, Umeå, SE

Key persons involved in early discussions
Göran Berglund, Göran Bergström, Björn Fagerberg, Ulf de Faire, Jan Engvall, Bo Hedblad, Christer Janson, Lars Johansson, Lars Lind, Claes-Göran Löfdahl, Fredrik Nyström, Anders Persson, Annika Rosengren, Stefan Söderberg, Anders Waldenström, Hans Wedel

Supportive Organization & Coordinating site
The Swedish Heart and Lung Foundation [2019-0012] is the main funder of SCAPIS. In addition to providing financial support, the Swedish Heart and Lung Foundation has facilitated this national collaborative project by assisting in arranging study meetings, providing travel support and meeting venues, and sharing their expertise in communicating science.

Swedish Heart and Lung Foundation
Kristina Sparreljung (secretary general), Staffan Josephsson (former secretary general), Mira Ernkvist, Bim Boberg, Amanda Skog Andreasson, Ebba Bergman, Anna Fredholm, Louise Fornander, Susanne Klofsten,

Christa Larsvall, Anna Sjöström, Sofia Swedenborg, Joanna Tingström, Caroline Waldenström Sylvén, Sofia Wirsén, Jan Nilsson, Anders Waldenström

Study operations

Operational Management Team
Eva Karin Anderberg, Anna Andreasson, Göran Bergström, Anna Beskow, Bim Boberg, Charlotta Elfström, Mira Ernkvist, Peter Hedman, Carl Johan Östgren

SCAPIS Office
Eva Karin Anderberg (coordinator), Kristina Levan, Åsa Odhagen Rosvall

National Project Managers
National Project Coordinator: Charlotta Elfström

Project Managers: Sven Anders Benjegård, Ebba Bergman, Martin Brandhagen, Sven Burman, Olivia Claesson, Louise Fornander, Anna Frick, Urban Gustafsson, Emma Larsson, Maria Matson Dzebo, Tina Noord, Klara Thorsson

Data Management
IT Project Lead: Tina Noord (main coordinator), Anita Adolfsson, Anna Andreasson

Product Owner and Product Specialist*:* Anna Frick, Christian Johansen, Emma Larsson

IT Development Team: *Solution Architects:* Christian Johansen, Tomas Snäckerström, Andreas Wallén*; System Developers:* Simon Fransson, Johan Fredin, Albin Willman

Study Data Management: Niklas Svensson

Image Data Storage: Emanuel Hillberg, Nasser Hosseini, Åke Marjamäki, Tomas Moberg

Accelerometry Data Management: Örjan Ekblom, Elin Ekblom-Bak

Local IT Support: *Gothenburg:* Anders Broman, Johan Fors, Roger Lampa; *Linköping:* Tomas Annerholm; *Malmö:* Pawel Gagol*; Umeå:* Gunnar Jonsson, Fredrik Lejon, Mattias Wennberg; *Uppsala:* Jörgen Anell, Martin Karlsson, José Conde Herrera

Head Project Statistician
Erik Lampa

Publication Support
Ebba Bergman, Louise Fornander, Sofia Swedenborg

Senior Scientific Editor
Rosie Perkins

Local Project Lead, Coordinators & Local Steering Committee

University of Gothenburg
Principal Investigators: Göran Bergström (PI), Annika Rosengren (co-PI); *Local Coordinator:* Caroline Schmidt; *Local Steering Committee Members:* Oskar Angerås, John Brandberg, Jan Hedner, Kjell Torén

Linköping University
Principal Investigators: Carl Johan Östgren (PI), Jan Engvall (co-PI), Carl-Johan Carlhäll (co-PI); *Local Coordinators*: Charlotte Brage, Elisabeth Logander; *Local Steering Committee Members*: Preben Bendtsen, Per Dannetun, Kjell Jansson, Magnus Janzon, Ditte Pehrsson-Lindell, Anders Persson, Mats Ulfendahl

Lund University
Principal Investigators: Gunnar Engström (PI), Margaretha Persson (co-PI); *Local Coordinator:* Margaretha Persson; *Local Steering Committee Members*: Lars Bååth, Joyce Carlsson, Arne Egesten, Olle Ekberg, David Erlinge, Isabel Goncalves, Anders Gottsäter, Bo Hedblad, Martin Magnusson, Olle Melander, Peter Nilsson, Eeva Piitulainen, Per Wollmer, Gerd Östling

Karolinska Institutet
Principal Investigators: Tomas Jernberg (PI), Magnus Sköld (co-PI); *Local Coordinators*: Liselotte Persson, Sven Burman, Erica Ottenblad, Teresa Sandvall, Cecilia Ström; *Local Steering Committee Members*: Kenneth Caidahl, Kerstin Cederlund, Maria Englund, Mats Eriksson, Maria Eriksson, Per Eriksson, Maria Mannila

Umeå University
Principal Investigators: Anders Blomberg (PI), Stefan Söderberg (co-PI); *Local Coordinator:* Kristin Ahlm, Anna Ramnemark; *Local Steering Committee Members*: Diana Berggren, Kjell Burman, Karl Gustav Forsberg, Magnus Hedström, Marlene Landström, Per Lindqvist, Cecilia Mattsson, Anna Ramnemark, Anette Sandström, Marie Strand, Jenny Åkerblom

Uppsala University
Principal Investigators: Johan Sundström (PI), Lars Lind (co-PI); *Local Coordinators:* Maria Storgärds; *Local Steering Committee Members:* Håkan Ahlström, Olov Duvernoy, Tove Fall, Emil Hagström, Stefan James, Christer Janson, Sune Larsson, Eva Lindberg, Andrei Malinovschi, Jonas Oldgren

Scientific Working Groups
Lars Lind (Chair of the Biomarker Group), Kjell Torén (Chair of the Lung and Pulmonary Group), Carl Johan Östgren (Chair of the Metabolism Group), Gunnar Engström (Chair of the Cohort Group), Jan Engvall (Chair of the Image Analysis Group)

Biobank Advisory Resources
Anna Beskow, Sonja Eaker Fält, Margaretha Persson, Gunnel Tybring

Quality Managers
Ultrasonography: Caroline Schmidt, Gerd Östling; *Computed Tomography (CT):* John Brandberg, Marit Johannesson, Helén Milde; *Coronary CT Angiography*: Lilian Henriksson; *Pulmonary Function and Vital Signs:* Annika Johansson, Cecilia Kennbäck

Study execution

Study Physicians
Gothenburg: Noraldeen Al-Dury, Anna Björk, Matilda Du Rietz, Hanna Eriksson, Magnus Hallor, Mattias Hallsten, Karin Hedman, Anna Jeppson, Josefina Robertsson, Sara Roos, Jessica Sjölund, Linda Thorvaldsson, Johan Thurell, Adam Zachrisson; *Linköping:* Eva Swahn (national convenor); *Malmö:* Erasmus Bachus, Klas Gränsbo, Viktor Hamrefors; *Stockholm:* Maria Mannila, Gundars Rasmanis, Izabella Zarea-Ganji; *Umeå:* Shariar Fezi Razi, Anja Isaksson, Lars Nilsson, Anette Sandström, Linn Skoglund; *Uppsala:* Johan Forsblad, Emil Hagström, Martin Sandelin

SCAPIS Site-Responsible Nurse/Team Lead
Gothenburg: Catherine Åhlund; *Linköping:* Elisabeth Logander; *Malmö:* Cecilia Kennbäck; *Stockholm:* Caroline Bäck, Pia Löf, Emma Stahre; *Umeå:* Kristin Ahlm; *Uppsala:* Aregash Tesfaldet

SCAPIS Site-Responsible Radiology Nurse
Gothenburg: Marit Johannesson, Helen Milde; *Linköping:* Lilian Henriksson; *Malmö:* Elisabeth Andersson; *Stockholm:* Aziza Adem; *Umeå:* Maria Lundbäck, David Wahllöf; *Uppsala:* Monica Segelsjö

Radiologists and cardiologists – Angiography

(in numerical order, starting with highest number of examinations/radiologist.)

Agneta Flinck (Gothenburg), Olov Duvernoy (Uppsala), Anders Hauggaard (Linköping), Raquel Themudo (Stockholm), Tanja Kero (Uppsala), Ellen Ostenfeld (Malmö), Lisa Ander Olsson (Malmö), Kerstin Cederlund (Stockholm), Catharina Adlercreutz (Malmö), Hans Lindgren (Umeå), Karen Sörensen (Umeå), Susann Skoog (Linköping), Erika Fagman (Gothenburg), Maria Kjellin (Linköping), Katharina Brehmer (Stockholm), Franciska Wikner (Umeå), Isabel Goncalves (Malmö), Hanna Markstad (Malmö), Anders Björkholm (Linköping), Caroline Berntsson (Gothenburg), Marcus Gjerde (Linköping), Elin Bacsovics Brolin (Stockholm), Louise Norlén (Stockholm), Johan Blomma (Linköping), Gunnar Wiklund (Linköping), Lisbeth Denbratt (Gothenburg), Adrian Pistea (Malmö), Kerstin Cederlund (Linköping), Artur Tomson (Stockholm), Margareta Klein (Stockholm), Gusten Nyberg (Linköping), Tomasz Baron (Uppsala), Ylva Gårdinger (Malmö), Viktor Hamrefors (Malmö), Sofia Olai (Linköping), Ming Chen (Stockholm)

Radiologists – Emphysema

(in numerical order, starting with highest number of examinations/radiologist.)

Johan Thurén (Uppsala), Marianne Boijsen (Gothenburg), Ulf Molin (Uppsala), Catharina Adlercreutz (Malmö), Anders Hauggaard (Linköping), Sören Strandberg (Stockholm), Lars Bååth (Malmö), Jenny Vikgren (Gothenburg), Kerstin Cederlund (Stockholm), Hans Lindgren (Umeå), Susann Skoog (Linköping), Lisa Ander Olsson (Malmö), Karen Sörensen (Umeå), Maria Kjellin (Linköping), Franciska Wikner (Umeå), Egon Wallier (Stockholm), Katharina Brehmer (Stockholm), Anders Björkholm (Linköping), Johan Blomma (Linköping), Hanna Markstad (Malmö), Gunnar Wiklund (Linköping), Bengt Gottfridsson (Gothenburg), Gusten Nyberg (Linköping), Andreas Malmqvist (Stockholm), Kerstin Cederlund (Linköping), Dariusz Slusarczyk (Malmö), Raquel Themudo (Stockholm), Bertil Larsson (Uppsala), Anna Kahn (Malmö), Rauni Rossi Norrlund (Gothenburg), Olov Duvernoy (Uppsala), Sofia Olai (Linköping), Charlotta Lidbjörk (Linköping), Åse Johnsson (Gothenburg), Tomas Hansen (Uppsala)

Other Personnel Categories of Importance

Biomedical analysts, radiology nurses, clinical and assistant nurses at the six sites.
Other persons involved in the governance, operation and execution of the project.

SCAPIS Site-Responsible Organization

Gothenburg: Gothia Forum; *Linköping:* Department of Clinical Physiology, Linköping University Hospital; *Malmö:* Clinical Research Unit, Department of Medicine, Skåne University Hospital, Malmö; *Stockholm:* Karolinska Trial Alliance (KTA); *Umeå:* Clinical Research Center, Region Västerbotten, Umeå; *Uppsala:* Uppsala Clinical Research Center/Uppsala Biobank, Uppsala University.

METHODS

Reproducibility and consistency of reading CCTA across readers and sites

Reproducibility of reading CCTA images was tested between two readers who repeated their assessment of 51 consecutive cases >4 weeks apart (**Table V** in the Supplement). In these readings, 10 of the 11 most proximal segments, but not segment 9 (first diagonal), were included.

To check for consistency of data across readers and sites, the relation between CCTA data and coronary artery calcium score (CACS) was examined given that CACS is considered to be less user-dependent than CCTA and there is a positive association between CACS and the extent of coronary atherosclerosis.[41,49] Frequency of the CCTA outcome (no atherosclerosis, 1-49% stenosis, ≥50% stenosis or calcium blooming) was plotted in relation to CACS categories for individual readers (**Figure II** in the Supplement) and for each site (**Figure III** in the Supplement).

Definitions of CCTA scores used to calculate burden of coronary atherosclerosis per subject

In the current paper, the following anatomical assumptions were made to define the three main coronary vessels:

Left anterior descending artery (LAD): (S5, S6, S7, S8, S9, S10 and S17)

Left circumflex artery (CXA): (S11, S12, S13, S14, S15, S18)

Right coronary artery (RCA): (S1, S2, S3, S4, S16)

One-vessel, two-vessel or three-vessel disease was then defined by a ≥50% stenosis in one, two or three of the above vessels.

Inverse probability for participation weighting (IPPW)

To test external validity, prevalence measures of coronary atherosclerosis were standardized the age-matched population in the SCAPIS catchment areas, which reflects the population that was invited to participate in SCAPIS or Sweden as a whole using sociodemographic register data and inverse probability for participation weighting (IPPW).[50, 51]

With assistance from Statistics Sweden, the SCAPIS participants were matched to each of the 5,985 demographic statistics areas (DSAs) in Sweden (1,925 of which are located within the SCAPIS catchment areas) using home addresses. DSAs are nested within municipalities and defined with respect to geography and population size, and their primary purpose is to monitor social segregation at a fine scale. The average population size of a DSA is ~300 (range: 0-719) for the population aged 50-64 years.

For each DSA, we asked Statistics Sweden to supply yearly data from 2013 to 2018 on the following covariates: (i) the median income per household, (ii) the percentage of immigrants born in non-western countries (Africa, Asia, Eastern Europe and South America), (iii) the proportion of individuals aged 50-64 with tertiary education, (iv) the unemployment rate for the population 20-64 years and, (v) population size by age group and sex. If a DSA was within the catchment areas for SCAPIS, we averaged these variables over the enrollment period for each site; for all other DSAs outside the SCAPIS catchment areas, we used mean values across the period 2013-2018. We used the data to characterize the SCAPIS participant dataset and two target populations of interest to SCAPIS: (1) the age-matched population in the SCAPIS catchment areas, which reflects the population that was invited to participate in SCAPIS, and (2) the entire Swedish population (aged 50-64 years).

We estimated two sets of inverse-odds-of-participation weights to standardize the SCAPIS sample to match each of these target populations on the distribution of DSA-level sociodemographic characteristics, as well as age group and sex. We stacked the two focal target populations data on top of the SCAPIS dataset and coded a participation dummy as “one” for participants and “zero” for the population.[51] We then used logistic generalized additive models with cubic splines to model the odds of belonging to the SCAPIS sample.[51]

Independent variables included in the models were age group, sex and the DSA-level characteristics. The inverse of the predicted odds of these models was then used to obtain the weighted estimates presented in **Table VII** in the Supplement.

Handling of calcium blooming

Defining the level of stenosis from calcified plaques is a challenge for CCTA and calcifications are known to overestimate the level of stenosis;[21,22] indeed, one study reported that only 29% of severely calcified segments were associated with significant obstructive disease.[21] If calcium content of the segment was severe and precluded judgement of level of stenosis, the segment was classified as having a calcium blooming artefact and, in the main analyses, the level of stenosis was set to 1-49%. In a sensitivity analysis presented in **Table IX** in the Supplement, the level of stenosis for a calcium blooming artefact was set to ≥50%.

SUPPLEMENTARY FIGURES

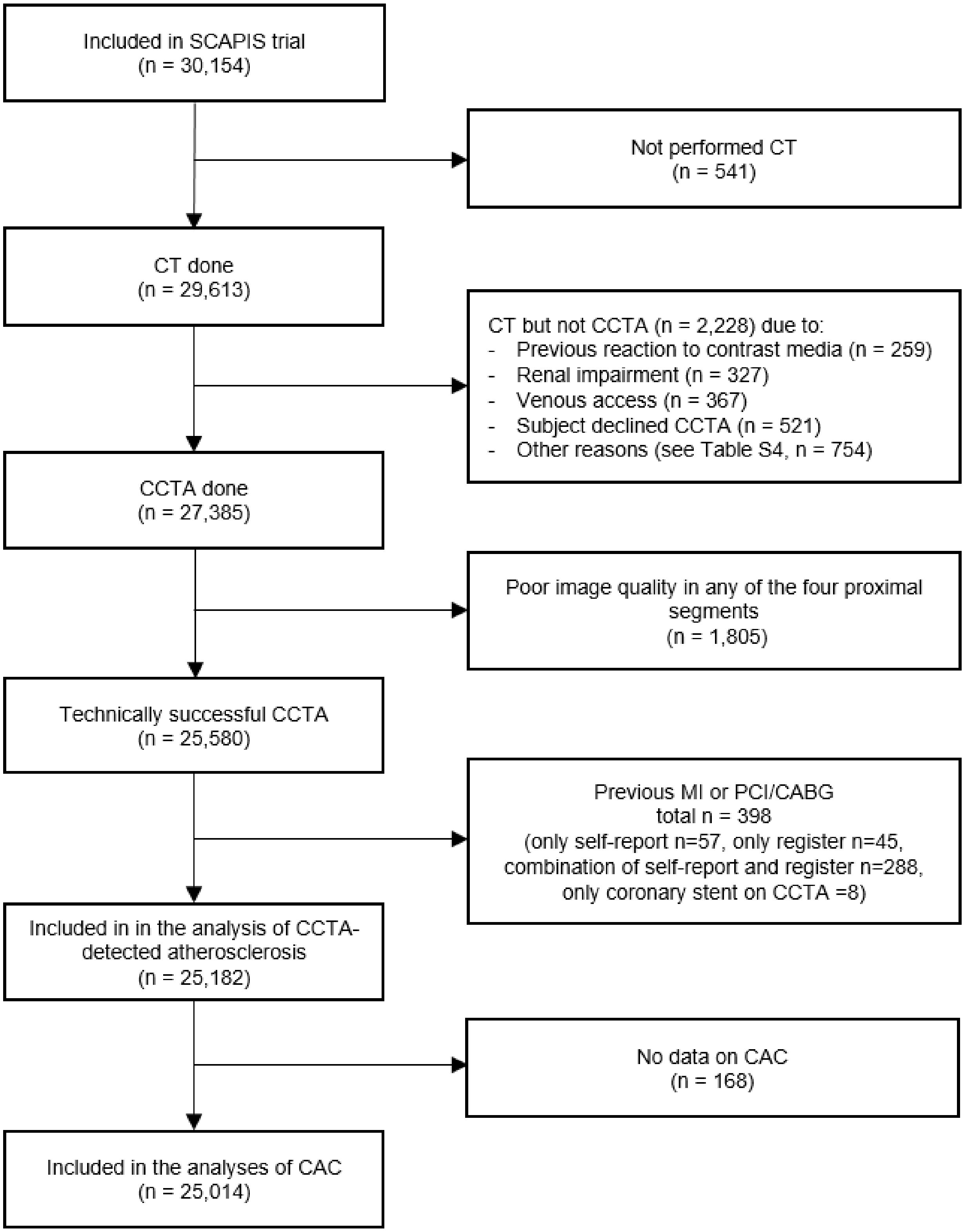

Supplemental Figure I: Flow diagram of study inclusion.

MI, myocardial infarction; PCI, percutaneous coronary intervention; CABG, coronary artery bypass grafting.

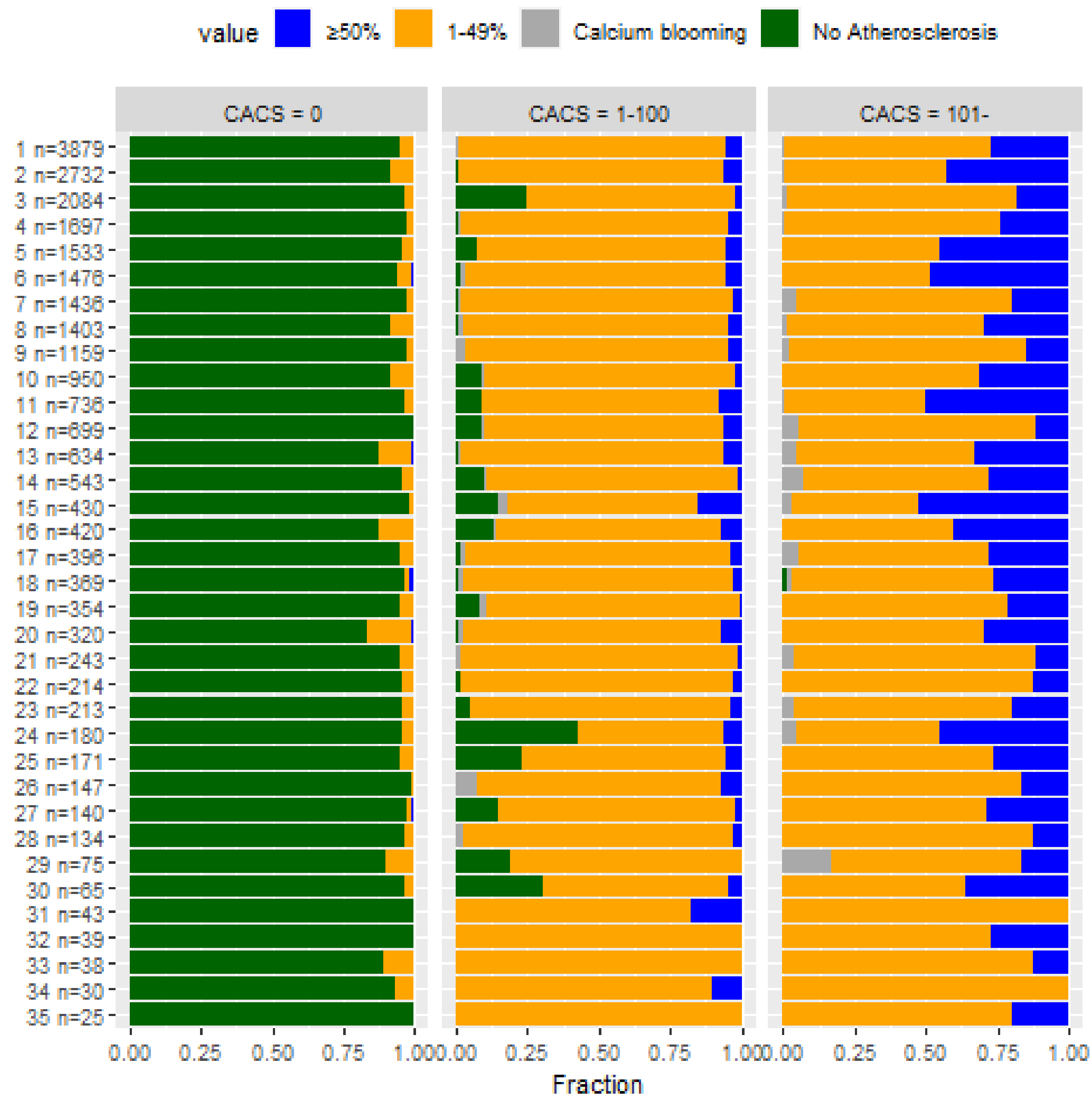

Supplemental Figure II: Atherosclerosis per subject for individual readers, stratified by CACS category.

Each subject was classified according to the highest segmental burden of coronary atherosclerosis (in increasing order: no atherosclerosis; calcium blooming; 1-49% stenosis; or ≥50% stenosis) in the 11 most proximal segments. Data reported from the 35 readers with at least 10 cases read (there were 37 readers in total) and n= 25,014 cases (CAC data were missing in 168 subjects).

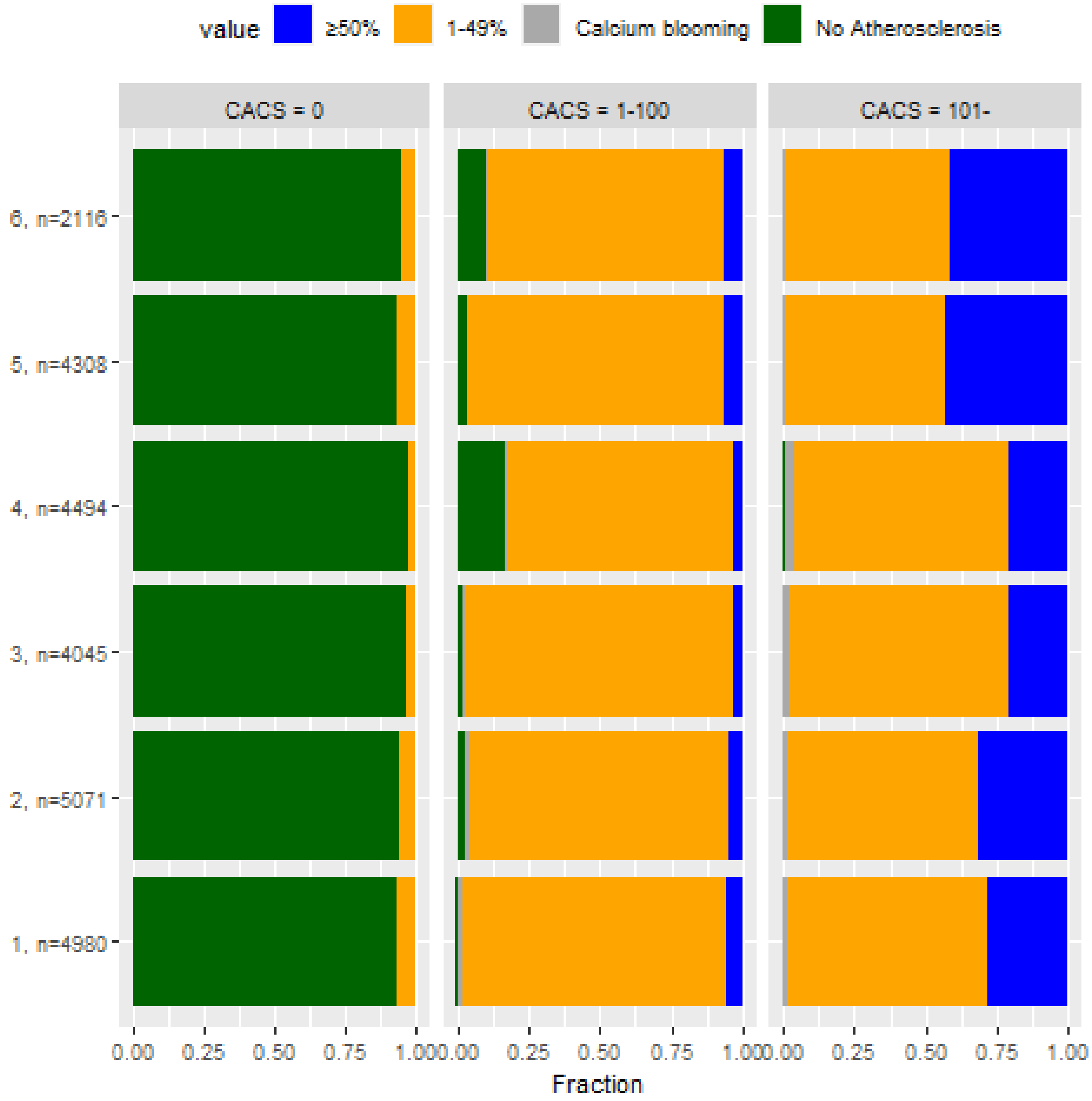

Supplemental Figure III: Atherosclerosis per subject for each site, stratified by CACS category.

Each subject was classified according to the highest segmental burden of coronary atherosclerosis (in increasing order: no atherosclerosis; calcium blooming; 1-49% stenosis; or ≥50% stenosis) in the 11 most proximal segments. Data reported per site and n= 25,014 (CACS data were missing in 168 subjects).

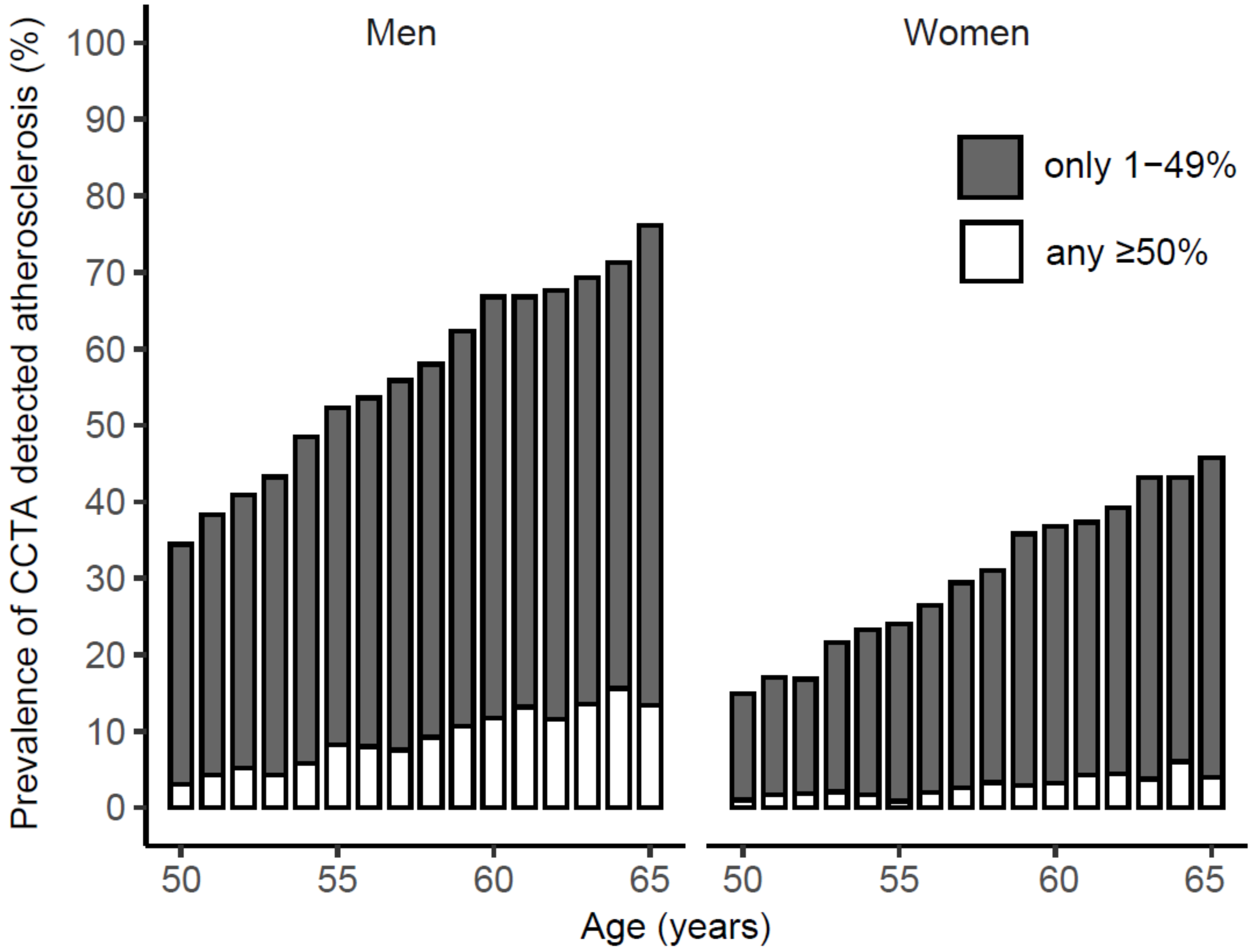

Supplemental Figure IV: Prevalence of CCTA-detected atherosclerosis by sex and age.

Prevalence of CCTA-detected coronary atherosclerosis and degree of stenosis in the SCAPIS cohort (n=25,182), divided by sex and age.

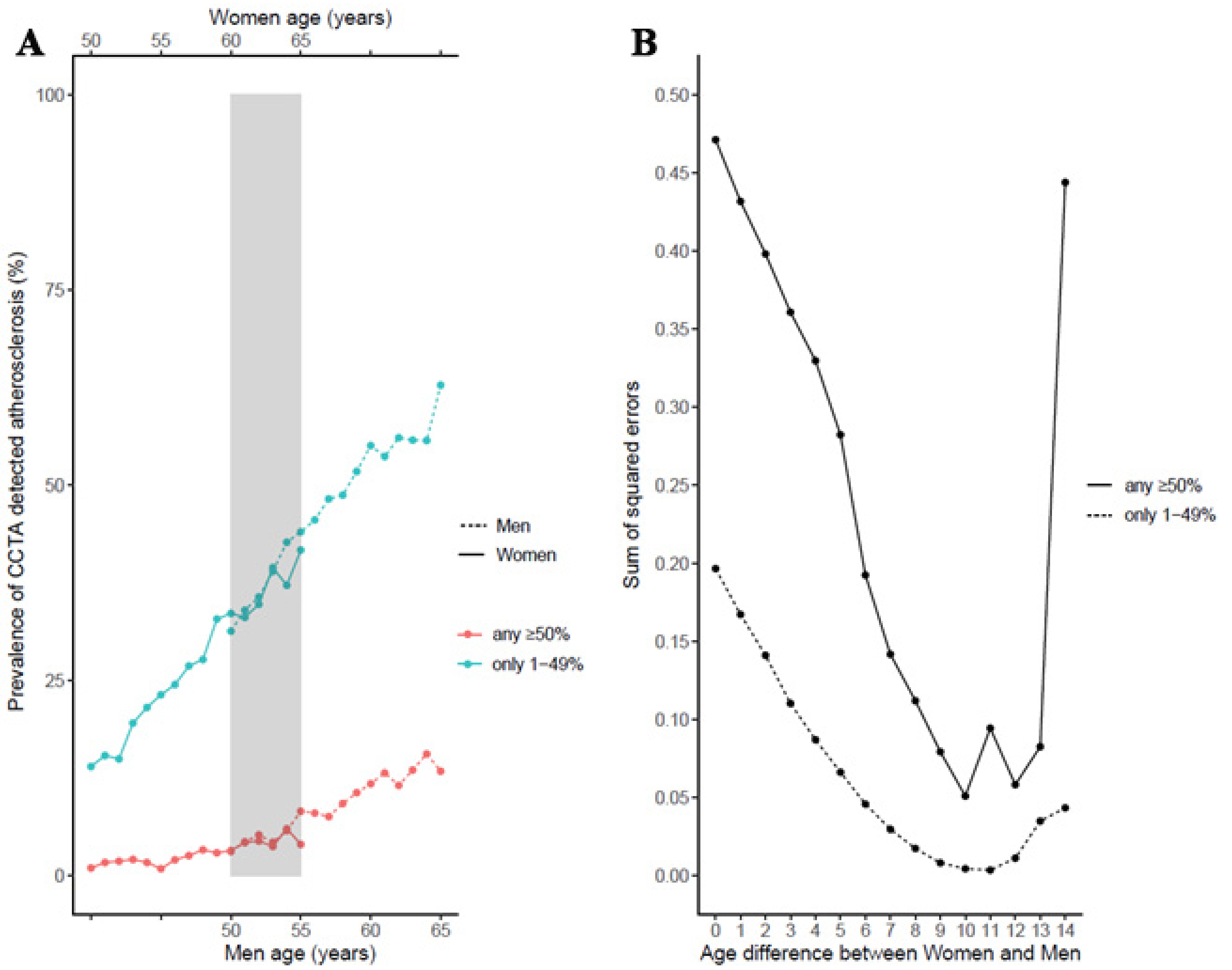

Supplemental Figure V: Modeling of delayed onset of coronary atherosclerosis in women.

The delayed onset of CCTA-detected atherosclerosis (any ≥50% stenosis and only 1-49% stenosis) in women compared to men was modeled by sliding the x-axis for women back and forth in relation to that of men (Panel A) in 1-year intervals, and identifying the minimal value of the sum of the squared differences between the annualized prevalence in women and men (Panel B).

SUPPLEMENTARY TABLES

Supplemental Table I. Characteristics of study participants in the full SCAPIS cohort.

Characteristic	Total	Men	Women
**Sample size — n**	30,154	14,646	15,508
Sociodemographics			
**Age — years**	57.5±4.4	57.5±4.4	57.5±4.3
**Education, university degree — n (%)**	13,218 (45.1)	5,769 (40.7)	7,449 (49.2)
**Employed — n (%)**	24,483 (81.2)	11,992 (81.9)	12,491 (80.5)
Anthropometry			
**Body mass index at age 20 — kg/m^2**	21.9±2.7	22.8±2.5	21.1±2.7
**Body mass index — kg/m^2**	27±4.5	27.5±4	26.5±4.9
**Waist circumference — cm**	94.4±13	99.8±11.2	89.4±12.5
**Behavior**			
Smoking status			
**Current smoker — n (%)**	3,728 (12.7)	1,798 (12.6)	1,930 (12.7)
**Former smoker — n (%)**	10,602 (36.0)	4,761 (33.4)	5,841 (38.5)
**Mean pack years**	7.5±12	7.5±12.6	7.4±11.3
**Duration of smoking — years**	11.8±15.6	11.2±15.5	12.5±15.7
Alcohol use			
**Once per month or less — n (%)**	7,198 (24.7)	2,909 (20.6)	4,289 (28.5)
**2-4 times per month — n (%)**	11,123 (38.1)	5,391 (38.2)	5,732 (38.0)
**More than once a week — n (%)**	10,865 (37.2)	5,816 (41.2)	5,049 (33.5)
Physical activity			
**Time spent sedentary — % of active time**	54±10.5	55.9±10.5	52.2±10.1
**Time in moderate or vigorous activity — % of active time**	6.4±3.4	6.6±3.6	6.2±3.2
Treatment			
**Cholesterol-lowering medication — (%)**	2,297 (7.6)	1,386 (9.5)	911 (5.9)
**Antihypertensive medication — n (%)**	5,768 (19.1)	3,034 (20.7)	2,734 (17.6)
**Diabetes medication — n (%)**	1,074 (3.6)	696 (4.8)	378 (2.4)
Blood pressure			
**Systolic blood pressure — mmHg**	126±17	129±16	123±18
**Diastolic blood pressure — mmHg**	78±11	79±10	77±11
Clinical chemistry			
**Total cholesterol — mmol/L**	5.5±1.1	5.3±1.1	5.6±1
**HDL cholesterol — mmol/L**	1.6±0.5	1.4±0.4	1.8±0.5
**LDL cholesterol — mmol/L**	3.4±1	3.4±1	3.5±1
**Triglycerides — mmol/L**	1.3±0.8	1.4±1	1.1±0.6
**Glucose — mmol/L**	5.8±1.1	6±1.2	5.6±1
**HbA1c — mmol/mol**	36.6±6.5	36.9±7.2	36.3±5.7
**High-sensitivity C-reactive protein — mg/L**	2.1±4.3	2.1±4.6	2.2±4.1
**Estimated GFR — ml/min/1.73 m^2**	85.1±12.1	85.7±11.7	84.5±12.4
Risk scores			
**SCORE — %**	1.4±1.3	2.1±1.4	0.7±0.6
**PCE risk score — %**	6.3±5.7	9.4±6.2	3.4±3.0
Prevalent cardiovascular disease			
**Stroke — n (%)**	465 (1.5)	263 (1.8)	202 (1.3)
**Peripheral artery disease — n (%)**	162 (0.5)	106 (0.7)	56 (0.4)
Heredity			
**Family history of premature myocardial infarction — n (%)**	2,012 (6.7)	883 (6.0)	1,129 (7.3)
**Family history of premature stroke — n (%)**	1,778 (5.9)	774 (5.3)	1,004 (6.5)

Plus-minus values are means ±SD. GFR, glomerular filtration rate. PCE, pooled cohort equation.

Supplemental Table II: Participation rate in SCAPIS per site, age and sex.

	Participants n (% of	Women n (% of participants)	Age groups, n (% of participants)		
			50-54 years	55-59 years	60-64 years
**Gothenburg**	6,265 (51.7)	3,276 (52.3)	2,058 (32.8)	2,128 (34.0)	2,079 (33.2)
**Linköping**	5,057 (58.0)	2,526 (50.0)	1,737 (34.3)	1,608 (31.8)	1,712 (33.9)
**Malmö**	6,251 (53.1)	3,322 (53.1)	2,057 (32.9)	2,081 (33.3)	2,113 (33.8)
**Stockholm**	5,038 (42.2)	2,517 (50.0)	1,749 (34.7)	1,664 (33.0)	1,625 (32.3)
**Umeå**	2,507 (54.5)	1,282 (51.1)	822 (32.8)	877 (35.0)	808 (32.2)
**Uppsala**	5,036 (46.8)	2,585 (51.3)	1,626 (32.3)	1,622 (32.2)	1,788 (35.5)
**Total**	**30,154 (50.3)**	**15,508 (51.4)**	**10,049 (33,3)**	**9,980 (33,1)**	**10,125 (33,6)**

59,909 subjects were randomly selected from the census register and contacted.

All percentages calculated within site.

Supplemental Table III: Characteristics of subjects who underwent CT but not CCTA.

Characteristic	SCAPIS	CT but not CCTA	CCTA sample
**Sample size — n**	30,154	2,228	25,182
Sociodemographics			
**Men — n (%)**	14,646 (48.6)	807 (36.2)	12,444
**Age — years**	57.5±4.4	58.2±4.3	57.4±4.3
**Education, university degree — n (%)**	13,218 (45.1)	880 (41.4)	11,263
**Employed — n (%)**	24,483 (81.2)	1,513 (67.9)	20,952
Anthropometry			
**Body mass index at age 20 — kg/m^2**	21.9±2.7	21.8±2.9	21.9±2.7
**Body mass index — kg/m^2**	27±4.5	27.6±5.6	26.8±4.3
**Waist circumference — cm**	94.4±13	95.1±15.2	94±12.6
**Behavior**			
Smoking status			
**Current smoker — n (%)**	3,728 (12.7)	309 (14.4)	3,079
**Former smoker — n (%)**	10,602 (36.0)	826 (38.4)	8,791
**Mean pack years**	7.5±12	8.7±13.1	7.2±11.7
**Duration of smoking — years**	11.8±15.6	13.5±16.5	11.6±15.4
Alcohol use			
**Once per month or less — n (%)**	7,198 (24.7)	764 (36.2)	5,649
**2-4 times per month — n (%)**	11,123 (38.1)	753 (35.6)	9,415
**More than once a week — n (%)**	10,865 (37.2)	596 (28.2)	9,452
Physical activity			
**Time spent sedentary — % of active time**	54±10.5	54.1±11.1	53.8±10.4
**Time in moderate or vigorous activity — % of active time**	6.4±3.4	6±3.5	6.4±3.4
Treatment			
**Cholesterol-lowering medication — (%)**	2,297 (7.6)	272 (12.2)	1,624 (6.4)
**Antihypertensive medication — n (%)**	5,768 (19.1)	636 (28.5)	4,437
**Diabetes medication — n (%)**	1,074 (3.6)	193 (8.7)	717 (2.8)
Blood pressure			
**Systolic blood pressure — mmHg**	126±17	126±18	126±17
**Diastolic blood pressure — mmHg**	78±11	77±11	78±10
Clinical chemistry			
**Total cholesterol — mmol/L**	5.5±1.1	5.5±1.1	5.5±1
**HDL cholesterol — mmol/L**	1.6±0.5	1.6±0.5	1.6±0.5
**LDL cholesterol — mmol/L**	3.4±1	3.4±1	3.5±1
**Triglycerides — mmol/L**	1.3±0.8	1.4±1.2	1.2±0.8
**Glucose — mmol/L**	5.8±1.1	6.1±1.8	5.7±1
**HbA1c — mmol/mol**	36.6±6.5	38.7±9.9	36.3±5.9
**High-sensitivity C-reactive protein — mg/L**	2.1±4.3	2.6±6.7	2.1±3.9
**Estimated GFR — ml/min/1.73 m^2**	85.1±12.1	80.5±17.3	85.4±11.5
Risk scores			
**SCORE — %**	1.4±1.3	1.3±1.3	1.4±1.3
**PCE risk score — %**	6.3±5.7	6.9±6.9	6.1±5.5
**Prevalent cardiovascular disease**			
**Stroke — n (%)**	465 (1.5)	59 (2.6)	343 (1.4)
**Peripheral artery disease — n (%)**	162 (0.5)	20 (0.9)	108 (0.4)
Heredity			
**Family history of premature myocardial infarction — n (%)**	2,012 (6.7)	153 (6.9)	1,619 (6.4)
**Family history of premature stroke — n (%)**	1,778 (5.9)	139 (6.2)	1,468 (5.8)

Plus–minus values are means ± SD. GFR, glomerular filtration rate. PCE, pooled cohort equation.

Supplemental Table IV: Reasons for not performing CCTA in subjects who underwent CT.

	Total	Men			Women		
		50-54 years	55-59 years	60-64 years	50-54 years	55-59 years	60-64 years
**Subjects who did not undergo CCTA**	2,228	214	260	329	397	493	531
**Previous reaction to contrast media — n (%)**	259 (11.6)	25 (11.7)	28 (10.8)	31 (9.4)	54 (13.6)	53 (10.8)	68 (12.8)
**Renal impairment — n (%)**	327 (14.7)	27 (12.6)	30 (11.5)	77 (23.4)	36 (9.1)	58 (11.8)	99 (18.6)
**Venous access — n (%)**	367 (16.5)	29 (13.6)	39 (15.0)	38 (11.6)	82 (20.7)	98 (19.9)	81 (15.3)
**Subject declined CCTA — n (%)**	521 (23.4)	52 (24.3)	53 (20.4)	49 (14.9)	108 (27.2)	129 (26.2)	128 (24.1)
**Other reasons, total — n (%)**	754 (33.8)	81 (37.9)	110 (42.3)	134 (40.7)	117 (29.5)	155 (31.4)	155 (29.2)
**Adverse reaction to drugs**	66 (8.8)	21 (25.9)	14 (12.7)	8 (6.0)	5 (4.3)	9 (5.8)	8 (5.2)
**Allergy, other than contrast**	63 (8.4)	8 (9.9)	8 (7.3)	8 (6.0)	7 (6.0)	20 (12.9)	12 (7.7)
**Logistics (creatinine)***	82 (10.9)	3 (3.7)	9 (8.2)	7 (5.2)	18 (15.4)	19 (12.3)	26 (16.8)
**Logistics (metformin)****	59 (7.8)	4 (4.9)	15 (13.6)	19 (14.2)	2 (1.7)	9 (5.8)	10 (6.5)
**Logistics (study organization)**	44 (5.8)	4 (4.9)	2 (1.8)	9 (6.7)	3 (2.6)	16 (10.3)	10 (6.5)
**Thyroid disease**	57 (7.6)	2 (2.5)	6 (5.5)	3 (2.2)	18 (15.4)	15 (9.7)	13 (8.4)
**Other diseases**	64 (8.5)	6 (7.4)	11 (10.0)	17 (12.7)	7 (6.0)	9 (5.8)	14 (9.0)
**Language difficulties**	90 (11.9)	13 (16.0)	13 (11.8)	15 (11.2)	20 (17.1)	15 (9.7)	14 (9.0)
**Miscellaneous or unclear**	229 (30.4)	20 (24.7)	32 (29.1)	48 (35.8)	37 (31.6)	43 (27.7)	48 (31.0)

*No access to p-creatinine before CCTA.

**Difficulties in postponing metformin treatment before CCTA.

Supplemental Table V: Missing data in participants in SCAPIS without established CHD who underwent successful CCTA (n=25,182).

Characteristic	Total
**Sociodemographics**	
**Age — n (%)**	0 (0)
**Education, university degree — n (%)**	589 (2.3)
**Employed — n (%)**	650 (2.6)
**Anthropometry**	
**Body mass index at age 20 — n (%)**	1,917 (7.6)
**Body mass index — n (%)**	0 (0.0)
**Waist circumference — n (%)**	8 (0.0)
**Behavior**	
***Smoking status (current/former)* — n (%)**	500 (2.0)
**Mean pack years — n (%)**	856 (3.4)
**Duration of smoking — n (%)**	46 (0.2)
***Alcohol use (complete AUDIT)* — n (%)**	804 (3.2)
***Physical activity (valid accelerometry)* — n (%)**	854 (3.4)
**Treatment**	
**Cholesterol-lowering medication — n (%)**	827 (3.3)
**Antihypertensive medication — n (%)**	825 (3.3)
**Diabetes medication — n (%)**	825 (3.3)
**Blood pressure**	
**Systolic blood pressure — n (%)**	6 (0.0)
**Diastolic blood pressure — n (%)**	4 (0.0)
**Clinical chemistry**	
**Total cholesterol — n (%)**	37 (0.1)
**HDL cholesterol — n (%)**	43 (0.2)
**LDL cholesterol — n (%)**	152 (0.6)
**Triglycerides — n (%)**	36 (0.1)
**Glucose — n (%)**	2,141 (8.5)
**HbA1c — n (%)**	83 (0.3)
**High-sensitivity C-reactive protein — n (%)**	36 (0.1)
**Estimated GFR — n (%)**	21 (0.1)
**Risk scores**	
**SCORE —n (%)**	41 (0.2)
**Pooled Cohort Equation — n (%)**	545 (2.2)
**Prevalent cardiovascular disease**	
**Stroke — n (%)**	815 (3.2)
**Peripheral artery disease — n (%)**	815 (3.2)
**Heredity**	
**Family history of premature myocardial infarction — n (%)**	596 (2.4)
**Family history of premature stroke — n (%)**	596 (2.4)

Supplemental Table VI. Inter- and intra-reader reproducibility for 10 coronary segments.

	Intra-agreement	Kappa	Inter-agreement	Kappa
**Segment (5 grade*)**	89	0.72 ± 0.03	89	0.67 ± 0.04
**Segment (dichotomous)****	98	0.88 ± 0.04	96	0.77 ± 0.05
**Five grade***				
**LAD**	92	0.85 ± 0.07	92	0.84 ± 0.07
**CXA**	90	0.63 ± 0.13	92	0.71 ± 0.11
**RCA**	98	0.92 ± 0.07	98	0.92 ± 0.08
**Dichotomous****				
**LAD**	98	0.96 ± 0.14	96	0.91 ± 0.14
**CXA**	98	0.91 ± 0.14	100	1.00 ± 0.14
**RCA**	98	0.90 ± 0.14	94	0.72 ± 0.12
**Subject (5 grade*)**	88	0.78 ± 0.16	86	0.78 ± 0.08
**Subject (dichotomous)****	96	0.91 ± 0.06	96	0.92 ± 0.06

Plus-minus values are means ±SD. Reading was done in 10 central segments (5, 6, 7, 11, 12, 13, 17, 1, 2, 3).

*The 5 grade analyses comprise: no atherosclerosis, 1-49% stenosis, ≥50% stenosis, calcium blooming, or technical artefact.

** The dichotomous analyses comprise: no atherosclerosis, any atherosclerosis (technical failures were coded as no atherosclerosis).

Supplemental Table VII: Technical failures and calcium blooming artefacts in the 11 central coronary vascular segments.

	Total		Men		Women	
	**Technical failures**	**Calcium blooming**	**Technical failures**	**Calcium blooming**	**Technical failures**	**Calcium blooming**
A. Subjects who completed CCTA (N=27,385)						
**Sample size — n**	27,385	27,385	13,618	13,618	13,767	13,767
**Subject level**	5,188 (18.9)	1,630 (6.0)	2,747 (20.2)	1,133 (8.3)	2,441 (17.7)	497 (3.6)
**Left coronary artery**	2,396 (8.7)	1,288 (4.7)	1,170 (8.6)	889 (6.5)	1,226 (8.9)	399 (2.9)
**Left main**	431 (1.6)	54 (0.2)	172 (1.3)	42 (0.3)	259 (1.9)	12 (0.1)
**Proximal LAD**	726 (2.7)	504 (1.8)	314 (2.3)	341 (2.5)	412 (3.0)	163 (1.2)
**Mid LAD**	1,136 (4.1)	725 (2.6)	560 (4.1)	502 (3.7)	576 (4.2)	223 (1.6)
**Ramus intermedius**	1,013 (3.7)	184 (0.7)	587 (4.3)	162 (1.2)	426 (3.1)	22 (0.2)
**First diagonal**	1,614 (6.1)	514 (1.9)	786 (6.0)	400 (3.0)	828 (6.2)	114 (0.8)
**Circumflex artery**	3,065 (11.2)	584 (2.1)	1,608 (11.8)	449 (3.3)	1,457 (10.6)	135 (1.0)
**Proximal circumflex**	899 (3.3)	253 (0.9)	406 (3.0)	180 (1.3)	493 (3.6)	73 (0.5)
**Obtuse marginal 1**	1,885 (6.9)	294 (1.1)	972 (7.1)	243 (1.8)	913 (6.6)	51 (0.4)
**Mid and distal circumflex**	2,099 (7.7)	202 (0.7)	1,066 (7.8)	166 (1.2)	1,033 (7.5)	36 (0.3)
**Right coronary artery**	2,607 (9.5)	432 (1.6)	1,270 (9.3)	307 (2.3)	1,337 (9.7)	125 (0.9)
**Proximal RCA**	928 (3.4)	190 (0.7)	433 (3.2)	138 (1.0)	495 (3.6)	52 (0.4)
**Mid RCA**	1,659 (6.1)	278 (1.0)	761 (5.6)	195 (1.4)	898 (6.5)	83 (0.6)
**Distal RCA**	1,389 (5.1)	157 (0.6)	673 (4.9)	132 (1.0)	716 (5.2)	25 (0.2)
B. Sample used to describe data on CCTA-detected atherosclerosis (n=25,182)						
**Sample size — n**	25,182	25,182	12,444	12,444	12,738	12,738
**Subject level**	3,206 (12.7)	1,309 (5.2)	1,742 (14.0)	892 (7.2)	1,464 (11.5)	417 (3.3)
**Left coronary artery**	1,103 (4.4)	1,041 (4.1)	577 (4.6)	705 (5.7)	526 (4.1)	336 (2.6)
**Left main**	0 (0.0)	30 (0.1)	0 (0.0)	23 (0.2)	0 (0.0)	7 (0.1)
**Proximal LAD**	0 (0.0)	389 (1.5)	0 (0.0)	250 (2.0)	0 (0.0)	139 (1.1)
**Mid LAD**	408 (1.6)	590 (2.3)	218 (1.8)	405 (3.3)	190 (1.5)	185 (1.5)
**Ramus intermedius**	612 (2.4)	134 (0.5)	384 (3.1)	116 (0.9)	228 (1.8)	18 (0.1)
**First diagonal**	842 (3.3)	403 (1.6)	430 (3.5)	309 (2.5)	412 (3.2)	94 (0.7)
**Circumflex artery**	1,702 (6.8)	441 (1.8)	938 (7.5)	331 (2.7)	764 (6.0)	110 (0.9)
**Proximal circumflex**	0 (0.0)	181 (0.7)	0 (0.0)	121 (1.0)	0 (0.0)	60 (0.5)
**Obtuse marginal 1**	964 (3.8)	227 (0.9)	537 (4.3)	186 (1.5)	427 (3.4)	41 (0.3)
**Mid and distal circumflex**	1,184 (4.7)	153 (0.6)	638 (5.1)	124 (1.0)	546 (4.3)	29 (0.2)
**Right coronary artery**	1,359 (5.4)	328 (1.3)	665 (5.3)	227 (1.8)	694 (5.4)	101 (0.8)
**Proximal RCA**	0 (0.0)	135 (0.5)	0 (0.0)	97 (0.8)	0 (0.0)	38 (0.3)
**Mid RCA**	873 (3.5)	211 (0.8)	404 (3.2)	145 (1.2)	469 (3.7)	66 (0.5)
**Distal RCA**	811 (3.2)	112 (0.4)	408 (3.3)	94 (0.8)	403 (3.2)	18 (0.1)

Data are n (%) of subjects with technical failures or calcium blooming.

Supplemental Table VIII: Characteristics of subjects with technical failures in any of the 4 most proximal segments.

Characteristic	Completed CCTA	Proximal segments technical failure	CCTA sample
**Sample size — n**	27,385	1,805	25,182
**CCTA protocol used**			
**1**	5,459 (19.9)	233 (12.9)	5,206 (20.7)
**2**	18,030 (65.8)	1,200 (66.5)	16,528 (65.6)
**3**	3,403 (12.4)	278 (15.4)	3,061 (12.2)
**4A**	336 (1.2)	50 (2.8)	278 (1.1)
**4B**	23 (0.1)	8 (0.4)	15 (0.1)
**5**	134 (0.5)	36 (2.0)	94 (0.4)
**Pre-medication CCTA**			
**β-blocker — n (%)**	19,929 (72.8)	1,181 (65.5)	18,502 (73.5)
**Nitroprusside — n (%)**	23,409 (85.5)	1,427 (79.1)	21,633 (85.9)
**Pulse at CCTA**	63.5±8.6	66.8±10.3	63.2±8.4
**Sociodemographics**			
**Men — n (%)**	13,618 (49.7)	863 (47.8)	12,444 (49.4)
**Age — years**	57.5±4.4	57.7±4.4	57.4±4.3
**Anthropometry**			
**Body mass index — kg/m^2**	26.9±4.4	28.4±5.2	26.8±4.3
**Waist circumference — cm**	94.4±12.7	98±13.9	94±12.6
**Behavior**			
Smoking status			
**Current smoker — n (%)**	3,341 (12.4)	203 (11.4)	3,079 (12.5)
**Former smoker — n (%)**	9,628 (35.9)	667 (37.6)	8,791 (35.6)
**Treatment**			
**Cholesterol-lowering medication — (%)**	1,999 (7.3)	204 (11.3)	1,624 (6.4)
**Antihypertensive medication — n (%)**	5,056 (18.5)	438 (24.3)	4,437 (17.6)
**Diabetes medication — n (%)**	868 (3.2)	100 (5.5)	717 (2.8)
**Blood pressure**			
**Systolic blood pressure — mmHg**	126±17	126±17	126±17
**Diastolic blood pressure — mmHg**	78±10	78±11	78±10
**Clinical chemistry**			
**Total cholesterol — mmol/L**	5.5±1.1	5.4±1.1	5.5±1
**HDL cholesterol — mmol/L**	1.6±0.5	1.6±0.5	1.6±0.5
**LDL cholesterol — mmol/L**	3.4±1	3.4±1	3.5±1
**Triglycerides — mmol/L**	1.2±0.8	1.3±0.9	1.2±0.8
**Glucose — mmol/L**	5.7±1.1	5.9±1.3	5.7±1
**HbA1c — mmol/mol**	36.4±6.1	37.5±8	36.3±5.9
**High-sensitivity C-reactive protein — mg/L**	2.1±3.9	2.4±4.1	2.1±3.9
**Estimated GFR — ml/min/1.73 m^2**	85.4±11.5	85.3±11.8	85.4±11.5
**Risk scores**			
**SCORE — %**	1.4±1.3	1.4±1.3	1.4±1.3
**PCE risk score — %**	6.3±5.6	6.8±6.3	6.1±5.5
**Prevalent cardiovascular disease**			
**Stroke — n (%)**	392 (1.4)	33 (1.8)	343 (1.4)
**Peripheral artery disease — n (%)**	141 (0.5)	23 (1.3)	108 (0.4)
**Heredity**			
**Family history of premature MI — n (%)**	1,822 (6.7)	148 (8.2)	1,619 (6.4)
**Family history of premature stroke — n**	1,619 (5.9)	117 (6.5)	1,468 (5.8)

Plus–minus values are means ±SD. GFR, glomerular filtration rate. PCE, pooled cohort equation.

Supplemental Table IX: Prevalence of atherosclerosis in the SCAPIS cohort after adjustment to age-matched populations using IPPW.

Characteristic	Unweighted*	Standardized to catchment area population 50-64 years	Standardized to national population 50-64 years
**No atherosclerosis**	57.9 (57.3-58.5)	57.9 (57.3-58.5)	57.2 (56.2-58.3)
**Any form of atherosclerosis**	42.1 (41.5-42.7)	42.1 (41.4-42.7)	42.7 (41.7-43.8)
**Any stenosis ≥50%**	5.2 (5.0-5.5)	5.3 (5.0-5.6)	6.2 (5.7-6.9)
**One vessel disease**	4.3 (4.0-4.5)	4.3 (4.0-4.6)	5.0 (4.5-5.5)
**Two vessel disease**	0.8 (0.7-0.9)	0.8 (0.7-0.9)	1.1 (0.8-1.4)
**Three vessel disease**	0.2 (0.1-0.3)	0.2 (0.1-0.2)	0.2 (0.1-0.3)
**Left main disease**	0.1 (0.1-0.2)	0.1 (0.1-0.2)	0.2 (0.1-0.3)
**Proximal LAD disease**	1.9 (1.7-2.0)	1.8 (1.7-2.0)	2.2 (1.8-2.6)
**Left main, proximal LAD or three-vessel disease**	1.9 (1.8-2.1)	1.9 (1.8-2.1)	2.3 (1.9-2.7)
**Segment involvement score ≥4%**	11.9 (11.5-12.3)	11.9 (11.5-12.3)	12.8 (12.1-13.6)
**Only non-calcified plaques**	2.4 (2.2-2.6)	2.5 (2.3-2.7)	2.5 (2.2-2.8)
**Any plaque non-calcified**	8.3 (8.0-8.7)	8.4 (8.0-8.8)	8.7 (8.1-9.4)
**Any non-calcified stenosis ≥50%**	1.2 (1.1-1.4)	1.3 (1.1-1.4)	1.3 (1.1-1.7)

Values are percentages with 95% robust binomial confidence intervals in parentheses that account for the sampling weights.

*Analysis sample (n = 25,182).

Supplemental Table X: Prevalence of atherosclerosis in the SCAPIS cohort when calcium blooming was defined as ≥50% stenosis.

Characteristic	Total	Men			Women		
		50-54 years	55-59 years	60-64 years	50-54 years	55-59 years	60-64 years
No atherosclerosis — n (%)	14,579 (57.9)	2,516 (58.8)	1,791 (43.4)	1,262 (31.3)	3,532 (81.2)	2,960 (70.7)	2,518 (59.9)
Any form of atherosclerosis — n (%)	10,603 (42.1)	1,762 (41.2)	2,337 (56.6)	2,776 (68.7)	816 (18.8)	1,229 (29.3)	1,683 (40.1)
Any stenosis ≥50% — n (%)	2,340 (9.3)	308 (7.2)	522 (12.6)	875 (21.7)	119 (2.7)	192 (4.6)	324 (7.7)
One vessel disease — n (%)	1,652 (6.6)	233 (5.4)	359 (8.7)	580 (14.4)	95 (2.2)	146 (3.5)	239 (5.7)
Two vessel disease — n (%)	446 (1.8)	52 (1.2)	107 (2.6)	179 (4.4)	20 (0.5)	31 (0.7)	57 (1.4)
Three vessel disease — n (%)	242 (1.0)	23 (0.5)	56 (1.4)	116 (2.9)	4 (0.1)	15 (0.4)	28 (0.7)
Left main disease — n (%)	65 (0.3)	5 (0.1)	12 (0.3)	31 (0.8)	2 (0.0)	5 (0.1)	10 (0.2)
Proximal LAD disease — n (%)	859 (3.4)	95 (2.2)	190 (4.6)	328 (8.1)	45 (1.0)	69 (1.6)	132 (3.1)
Left main, proximal LAD or three-vessel disease — n (%)	935 (3.7)	107 (2.5)	207 (5.0)	359 (8.9)	46 (1.1)	78 (1.9)	138 (3.3)
Segment involvement score ≥4 — n (%)	3,003 (11.9)	424 (9.9)	753 (18.2)	1,182 (29.3)	93 (2.1)	201 (4.8)	350 (8.3)
Only non-calcified plaques — n (%)	607 (2.4)	117 (2.7)	107 (2.6)	94 (2.3)	100 (2.3)	96 (2.3)	93 (2.2)
Any plaque non-calcified — n (%)	2,102 (8.3)	359 (8.4)	492 (11.9)	575 (14.2)	160 (3.7)	224 (5.3)	292 (7.0)
Any non-calcified stenosis ≥50% — n (%)	315 (1.3)	42 (1.0)	80 (1.9)	110 (2.7)	16 (0.4)	24 (0.6)	43 (1.0)

Supplemental Table XI. Distribution of CCTA-detected atherosclerosis in the 11 most proximal coronary segments in men and women divided by age group in the SCAPIS cohort (n=25,182).

50-54 years of age

Men (denominator 4,278)				Women (denominator 4,348)			
**Segment**	**Any stenosis ≥50%**	**Only stenosis 1-49%**	**Any form of atherosclerosis**	**Segment**	**Any stenosis ≥50%**	**Only stenosis 1-49%**	**Any form of atherosclerosis**
**s01**	14 (0.3%)	376 (8.8%)	390 (9.1%)	**s01**	2 (0%)	127 (2.9%)	129 (3%)
**s02**	17 (0.4%)	393 (9.2%)	410 (9.6%)	**s02**	6 (0.1%)	129 (3%)	135 (3.1%)
**s03**	7 (0.2%)	238 (5.6%)	245 (5.7%)	**s03**	2 (0%)	62 (1.4%)	64 (1.5%)
**s04**	6 (0.1%)	6 (0.1%)	12 (0.3%)	**s04**	0 (0%)	0 (0%)	0 (0%)
**s05**	4 (0.1%)	306 (7.2%)	310 (7.2%)	**s05**	1 (0%)	118 (2.7%)	119 (2.7%)
**s06**	62 (1.4%)	1129 (26.4%)	1191 (27.8%)	**s06**	18 (0.4%)	497 (11.4%)	515 (11.8%)
**s07**	80 (1.9%)	812 (19%)	892 (20.9%)	**s07**	19 (0.4%)	288 (6.6%)	307 (7.1%)
**s08**	4 (0.1%)	5 (0.1%)	9 (0.2%)	**s08**	0 (0%)	2 (0%)	2 (0%)
**s09**	30 (0.7%)	225 (5.3%)	255 (6%)	**s09**	6 (0.1%)	59 (1.4%)	65 (1.5%)
**s10**	3 (0.1%)	9 (0.2%)	12 (0.3%)	**s10**	0 (0%)	5 (0.1%)	5 (0.1%)
**s11**	11 (0.3%)	382 (8.9%)	393 (9.2%)	**s11**	2 (0%)	130 (3%)	132 (3%)
**s12**	8 (0.2%)	149 (3.5%)	157 (3.7%)	**s12**	0 (0%)	38 (0.9%)	38 (0.9%)
**s13**	16 (0.4%)	133 (3.1%)	149 (3.5%)	**s13**	1 (0%)	31 (0.7%)	32 (0.7%)
**s14**	1 (0%)	6 (0.1%)	7 (0.2%)	**s14**	0 (0%)	2 (0%)	2 (0%)
**s15**	0 (0%)	1 (0%)	1 (0%)	**s15**	0 (0%)	1 (0%)	1 (0%)
**s16**	1 (0%)	1 (0%)	2 (0%)	**s16**	0 (0%)	0 (0%)	0 (0%)
**s17**	5 (0.1%)	105 (2.5%)	110 (2.6%)	**s17**	4 (0.1%)	13 (0.3%)	17 (0.4%)
**s18**	0 (0%)	0 (0%)	0 (0%)	**s18**	0 (0%)	0 (0%)	0 (0%)

55-59 years of age

Men (denominator 4,128)				Women (denominator 4,189)			
**Segment**	**Any stenosis ≥50%**	**Only stenosis 1-49%**	**Any form of atherosclerosis**	**Segment**	**Any stenosis ≥50%**	**Only stenosis 1-49%**	**Any form of atherosclerosis**
**s01**	37 (0.9%)	629 (15.2%)	666 (16.1%)	**s01**	5 (0.1%)	252 (6%)	257 (6.1%)
**s02**	43 (1%)	572 (13.9%)	615 (14.9%)	**s02**	15 (0.4%)	210 (5%)	225 (5.4%)
**s03**	28 (0.7%)	366 (8.9%)	394 (9.5%)	**s03**	3 (0.1%)	102 (2.4%)	105 (2.5%)
**s04**	6 (0.1%)	13 (0.3%)	19 (0.5%)	**s04**	1 (0%)	3 (0.1%)	4 (0.1%)
**s05**	6 (0.1%)	558 (13.5%)	564 (13.7%)	**s05**	3 (0.1%)	216 (5.2%)	219 (5.2%)
**s06**	111 (2.7%)	1585 (38.4%)	1696 (41.1%)	**s06**	29 (0.7%)	790 (18.9%)	819 (19.6%)

**s07**	137 (3.3%)	1111 (26.9%)	1248 (30.2%)	**s07**	31 (0.7%)	474 (11.3%)	505 (12.1%)
**s08**	5 (0.1%)	10 (0.2%)	15 (0.4%)	**s08**	0 (0%)	2 (0%)	2 (0%)
**s09**	58 (1.4%)	349 (8.5%)	407 (9.9%)	**s09**	6 (0.1%)	110 (2.6%)	116 (2.8%)
**s10**	6 (0.1%)	13 (0.3%)	19 (0.5%)	**s10**	1 (0%)	6 (0.1%)	7 (0.2%)
**s11**	27 (0.7%)	552 (13.4%)	579 (14%)	**s11**	6 (0.1%)	249 (5.9%)	255 (6.1%)
**s12**	25 (0.6%)	244 (5.9%)	269 (6.5%)	**s12**	2 (0%)	61 (1.5%)	63 (1.5%)
**s13**	17 (0.4%)	212 (5.1%)	229 (5.5%)	**s13**	4 (0.1%)	57 (1.4%)	61 (1.5%)
**s14**	3 (0.1%)	7 (0.2%)	10 (0.2%)	**s14**	0 (0%)	2 (0%)	2 (0%)
**s15**	1 (0%)	2 (0%)	3 (0.1%)	**s15**	1 (0%)	1 (0%)	2 (0%)
**s16**	3 (0.1%)	6 (0.1%)	9 (0.2%)	**s16**	0 (0%)	2 (0%)	2 (0%)
**s17**	26 (0.6%)	142 (3.4%)	168 (4.1%)	**s17**	1 (0%)	28 (0.7%)	29 (0.7%)
**s18**	0 (0%)	0 (0%)	0 (0%)	**s18**	0 (0%)	1 (0%)	1 (0%)
				**60-65 years of age**			
	**Men (denominator 4,038)**				**Women (denominator 4,200)**		
**Segment**	**Any stenosis ≥50%**	**Only stenosis 1-49%**	**Any form of atherosclerosis**	**Segment**	**Any stenosis ≥50%**	**Only stenosis 1-49%**	**Any form of atherosclerosis**
**s01**	53 (1.3%)	955 (23.7%)	1008 (25%)	**s01**	20 (0.5%)	382 (9.1%)	402 (9.6%)
**s02**	62 (1.5%)	861 (21.3%)	923 (22.9%)	**s02**	16 (0.4%)	357 (8.5%)	373 (8.9%)
**s03**	36 (0.9%)	580 (14.4%)	616 (15.3%)	**s03**	8 (0.2%)	156 (3.7%)	164 (3.9%)
**s04**	7 (0.2%)	21 (0.5%)	28 (0.7%)	**s04**	4 (0.1%)	6 (0.1%)	10 (0.2%)
**s05**	15 (0.4%)	848 (21%)	863 (21.4%)	**s05**	6 (0.1%)	342 (8.1%)	348 (8.3%)
**s06**	176 (4.4%)	1962 (48.6%)	2138 (52.9%)	**s06**	50 (1.2%)	1157 (27.5%)	1207 (28.7%)
**s07**	223 (5.5%)	1476 (36.6%)	1699 (42.1%)	**s07**	53 (1.3%)	659 (15.7%)	712 (17%)
**s08**	11 (0.3%)	16 (0.4%)	27 (0.7%)	**s08**	0 (0%)	4 (0.1%)	4 (0.1%)
**s09**	88 (2.2%)	507 (12.6%)	595 (14.7%)	**s09**	19 (0.5%)	152 (3.6%)	171 (4.1%)
**s10**	11 (0.3%)	33 (0.8%)	44 (1.1%)	**s10**	4 (0.1%)	10 (0.2%)	14 (0.3%)
**s11**	48 (1.2%)	918 (22.7%)	966 (23.9%)	**s11**	9 (0.2%)	398 (9.5%)	407 (9.7%)
**s12**	39 (1%)	397 (9.8%)	436 (10.8%)	**s12**	4 (0.1%)	113 (2.7%)	117 (2.8%)
**s13**	25 (0.6%)	320 (7.9%)	345 (8.5%)	**s13**	6 (0.1%)	106 (2.5%)	112 (2.7%)
**s14**	5 (0.1%)	7 (0.2%)	12 (0.3%)	**s14**	0 (0%)	3 (0.1%)	3 (0.1%)
**s15**	0 (0%)	3 (0.1%)	3 (0.1%)	**s15**	0 (0%)	0 (0%)	0 (0%)
**s16**	4 (0.1%)	4 (0.1%)	8 (0.2%)	**s16**	1 (0%)	4 (0.1%)	5 (0.1%)
**s17**	27 (0.7%)	219 (5.4%)	246 (6.1%)	**s17**	3 (0.1%)	53 (1.3%)	56 (1.3%)
**s18**	1 (0%)	2 (0%)	3 (0.1%)	**s18**	0 (0%)	0 (0%)	0 (0%)

Supplemental Table XII: Characteristics of participants in SCAPIS without established CHD who underwent both successful CCTA and CAC scoring (n=25,014), divided by zero or positive CAC and absence or presence of CCTA-detected atherosclerosis.

	CAC = 0		CAC >0	
**Characteristic**	**CCTA-**	**CCTA+**	**CCTA-**	**CCTA+**
**Sample size — n**	14,139	818	367	9690
**Sociodemographics**				
**Sex, women — n (%)**	8,807 (62.3)	400 (48.9)	154 (42.0)	3,305 (34.1)
**Age — years**	56.6±4.3	57.1±4.3	57.4±4.3	58.6±4.2
**Education, university degree — n (%)**	6,757 (48.8)	363 (45.3)	135 (38.4)	3,942 (41.8)
**Employed — n (%)**	12,122 (85.7)	666 (81.4)	297 (80.9)	7725 (79.7)
**Anthropometry**				
**Body mass index at age 20 — kg/m^2**	21.6±2.6	21.9±2.7	22.3±2.6	22.4±2.7
**Body mass index — kg/m^2**	26.3±4.2	27.4±4.1	27.7±4.4	27.4±4.3
**Waist circumference — cm**	91.6±12.3	95.5±11.8	96.4±12.5	97.2±12.4
**Behavior**				
Smoking status				
**Current smoker — n (%)**	1,445 (10.4)	105 (13.1)	36 (10.2)	1,482 (15.7)
**Former smoker — n (%)**	4,613 (33.2)	273 (34.0)	119 (33.8)	3,716 (39.2)
**Mean pack years**	5.7±10.1	7.2±11.7	6.4±11.7	9.4±13.4
**Duration of smoking — years**	9.8±14.2	11.6±15.4	10.1±15.1	14.2±16.7
Alcohol use				
**Once per month or less — n (%)**	3,246 (23.5)	226 (28.3)	91 (26.0)	2,054 (21.9)
**2-4 times per month — n (%)**	5,349 (38.7)	283 (35.4)	150 (42.9)	3,564 (38.0)
**More than once a week — n (%)**	5,234 (37.8)	290 (36.3)	109 (31.1)	3,765 (40.1)
Physical activity				
**Time spent sedentary — % of active time**	53.3±10.2	55.0±10.4	55.8±10.1	54.4±10.5
**Time in moderate or vigorous activity — % of active time**	6.5±3.3	6.3±3.6	6.3±3.2	6.4±3.5
**Treatment**				
**Cholesterol-lowering medication — (%)**	498 (3.5)	36 (4.4)	24 (6.5)	1,053 (10.9)
**Antihypertensive medication — n (%)**	1,736 (12.3)	154 (18.8)	71 (19.3)	2,441 (25.2)
**Diabetes medication — n (%)**	215 (1.5)	19 (2.3)	11 (3.0)	469 (4.8)
**Blood pressure**				
**Systolic blood pressure — mmHg**	123±17	128±17	131±17	129±16

**Diastolic blood pressure — mmHg**	76±10	79±11	82±11	79±10
**Clinical chemistry**				
**Total cholesterol — mmol/L**	5.5±1.0	5.6±1.1	5.6±1.1	5.6±1.1
**HDL cholesterol — mmol/L**	1.7±0.5	1.6±0.5	1.5±0.5	1.5±0.5
**LDL cholesterol — mmol/L**	3.4±0.9	3.6±0.9	3.5±0.9	3.6±1.0
**Triglycerides — mmol/L**	1.1±0.7	1.3±0.8	1.3±0.7	1.4±0.9
**Glucose — mmol/L**	5.6±0.8	5.7±0.9	5.7±0.8	5.9±1.3
**HbA1c — mmol/mol**	35.6±4.7	36.2±6.3	36.3±5.6	37.2±7.1
**High-sensitivity C-reactive protein — mg/L**	1.9±3.5	2.3±4.4	1.9±2.1	2.2±4.4
**Estimated GFR — ml/min/1.73 m^2**	85±12	86±11	84±11	86±11
**Risk scores**				
**SCORE — %**	1.0±1.0	1.4±1.3	1.6±1. 5	1.9±1.5
**PCE — %**	4.6±4.1	6.5±5.5	6.9±5.3	8.6±6.3
**Prevalent cardiovascular disease**				
**Stroke — n (%)**	147 (1.0)	11 (1.3)	2 (0.5)	181 (1.9)
**Peripheral artery disease — n (%)**	31 (0.2)	1 (0.1)	0 (0.0)	76 (0.8)
**Heredity**				
**Family history of premature myocardial infarction — n (%)**	799 (5.7)	51 (6.2)	27 (7.4)	731 (7.5)
**Family history of premature stroke — n (%)**	762 (5.4)	51 (6.2)	29 (7.9)	616 (6.4)

Plus-minus values are means ±SD. GFR, glomerular filtration rate. PCE, pooled cohort equation.

Supplemental Table XIII. Odds ratios (95% confidence intervals) for risk factors associated with discrepancies between CCTA-detected atherosclerosis and CAC scores in participants in SCAPIS without established CHD who underwent both successful CCTA and CAC scoring.

Characteristic	CCTA+ (n=818) in the population with zero CAC (n=14,957)	CAC >0 (n=367) in the population without CCTA-detected atherosclerosis (n=14,506)
**Sample size (complete case analyses) — n**	14,679	14,228
Sociodemographics		
**Sex (male)**	1.48 (1.259, 1.739)	1.902 (1.494, 2.421)
**Age (per year)**	1.021 (1.004, 1.039)	1.034 (1.007, 1.06)
Anthropometry		
**Obesity (body mass index >30)**	1.252 (1.045, 1.5)	1.331 (1.025, 1.73)
Smoking status		
**Current smoker**	1.264 (1.019, 1.568)	0.993 (0.697, 1.413)
Treatment		
**Cholesterol-lowering medication**	1.074 (0.744, 1.55)	1.522 (0.961, 2.41)
**Antihypertensive medication**	1.383 (1.135, 1.687)	1.222 (0.914, 1.635)
**Diabetes, self report**	0.946 (0.665, 1.345)	1.247 (0.801, 1.941)
Blood pressure		
**Systolic blood pressure (per mmHg)**	1.01 (1.005, 1.014)	1.018 (1.011, 1.024)
Clinical chemistry		
**Total cholesterol (per mmol/L)**	1.222 (1.138, 1.313)	1.159 (1.04, 1.29)
**HDL cholesterol (per mmol/L)**	0.642 (0.536, 0.768)	0.674 (0.514, 0.884)

Odds ratios are calculated per unit change for continuous variables.
