# Supplementary figures and images for: Prevalence of Subclinical Coronary Artery Atherosclerosis in the General Population
Source: Circulation. 2021 Sep 21;144(12):916–29. doi: 10.1161/CIRCULATIONAHA.121.055340 (PMC8448414; doi:10.1161/CIRCULATIONAHA.121.055340)

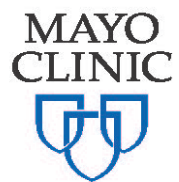

200 First Street SW  
Rochester, MN 55905  
(507) 284-3335  
**Scientific Publications**

*Rosemary Perry*

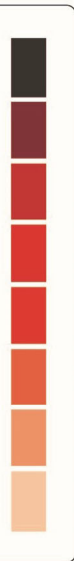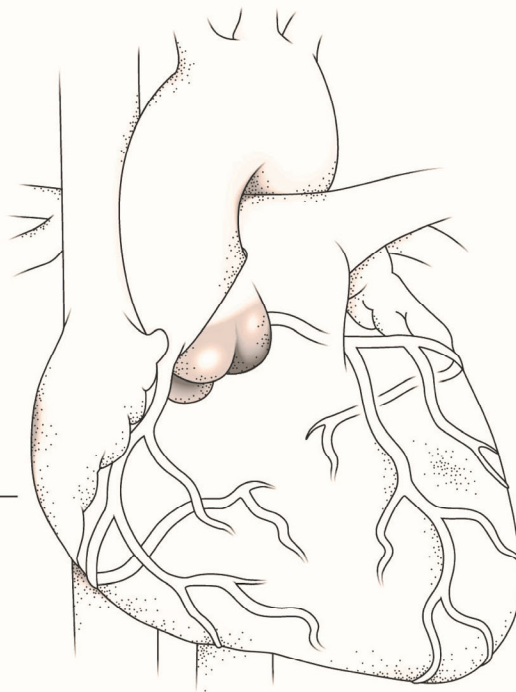

**MEN**

**WOMEN**

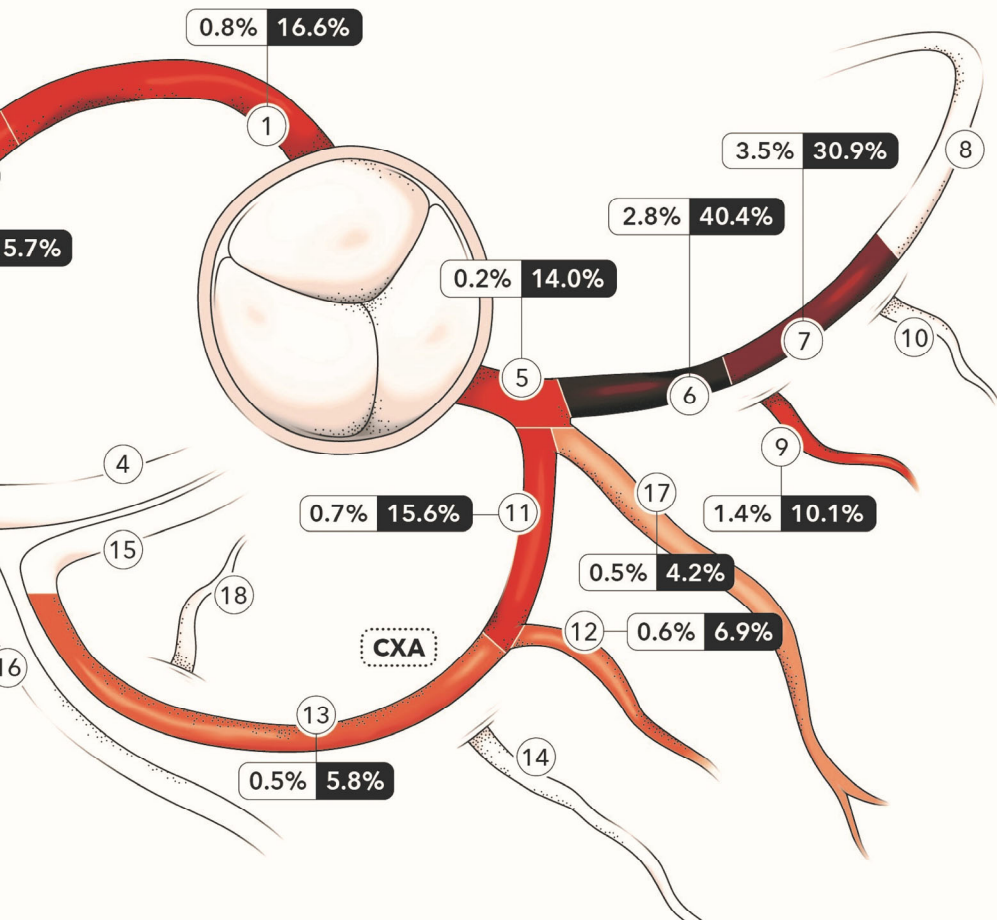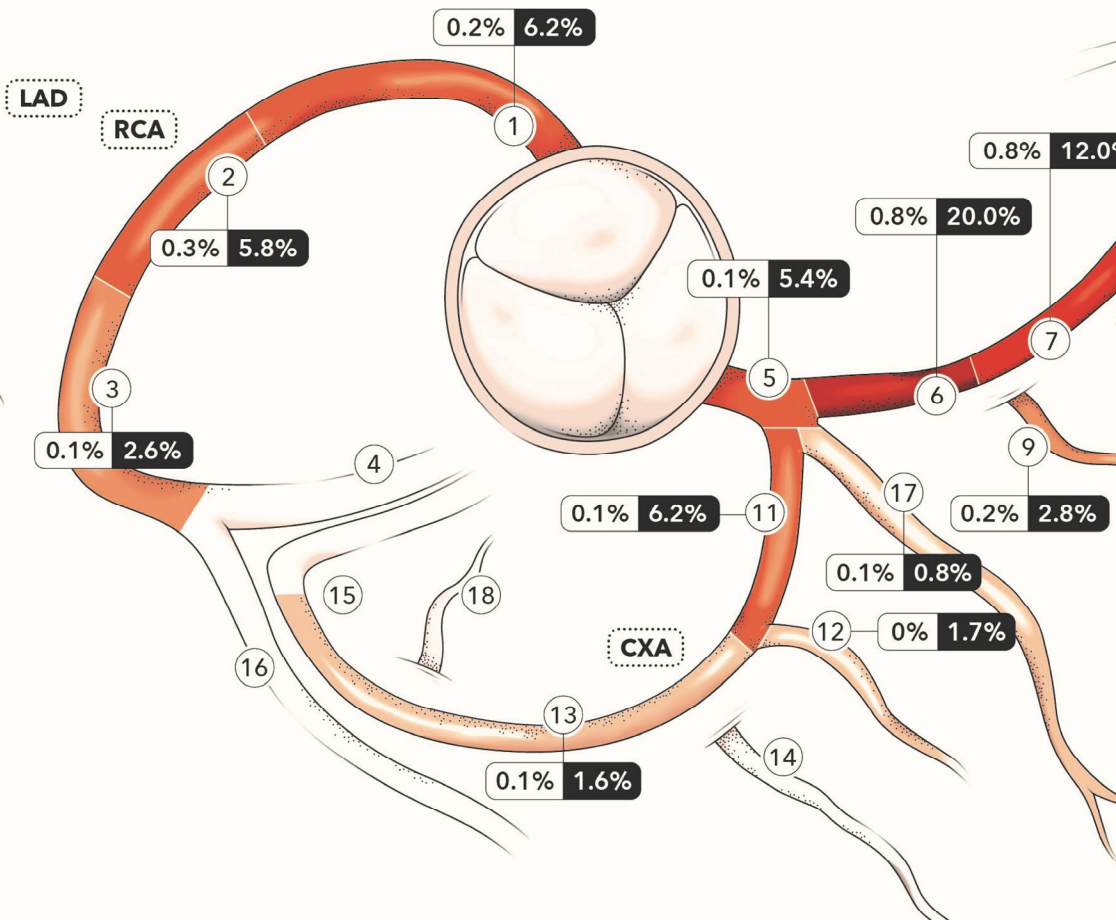

Supplement: Supplementary file 2 [file cir-144-916-s002.pdf]
